# Patterns of psychiatric healthcare use during pandemic times among boys and girls with pre-existing diagnoses: a Norwegian nationwide primary and specialist healthcare registry study

**DOI:** 10.1186/s12888-024-06422-7

**Published:** 2025-01-22

**Authors:** Ingunn Olea Lund, Pia Jensen, Christian Madsen, Lars Johan Hauge, Alexandra Havdahl, Anne Reneflot, Ragnhild Brandlistuen, Helga Ask

**Affiliations:** 1 PsychGen Center for Genetic Epidemiology and Mental Health, Norwegian Institute of Public Health, PO Box 222, Skøyen, Oslo, 0213 Norway; 2 Department of Child Health and Development, Norwegian Institute of Public Health, Oslo, Norway; 3 Department of Psychology, University of Oslo, Oslo, Norway; 4 Centre for Disease Burden, Norwegian Institute of Public Health, Bergen, Norway; 5 Department of Mental Health and Suicide, Norwegian Institute of Public Health, Oslo, Norway; 6 Nic Waals Institute, Lovisenberg Diaconal Hospital, Oslo, Norway; 7 Department of Psychology, PROMENTA, University of Oslo, Oslo, Norway

**Keywords:** Children, Adolescents, Mental disorders, Primary health care, Specialist health care, Health registry, COVID-19, Time trends

## Abstract

**Background:**

The COVID-19 pandemic introduced complexities that were likely more demanding for some groups, such as children and adolescents, and especially those with pre-existing mental health diagnoses. This study examines long-term patterns of psychiatric healthcare use among this vulnerable group, providing insights into shifts in psychiatric healthcare use during a global health crisis.

**Methods:**

We use data from the primary and specialist healthcare registries available from the Norwegian emergency preparedness register for COVID-19 (Beredt C19) to estimate patterns of psychiatric healthcare use. The data spans 2017 to 2022, covering children and adolescents aged 6–19. We identified young people with recent diagnoses of mental health conditions and compared weekly consultation volumes before and during the pandemic for mental health conditions overall and the following diagnostic or symptom categories separately: anxiety/depression and Attention-Deficit Hyperactivity Disorders (ADHD). Analyses were stratified by gender.

**Results:**

There was a consistent trend of lower-than-predicted weekly healthcare consultations among young people with pre-existing mental health diagnoses in both primary and specialist healthcare during the pandemic. The reduction was more pronounced in later follow-up periods. The study highlights gender disparities, with boys experiencing more notable declines in healthcare consultations, especially in specialist care. Additionally, the time trends varied across different diagnostic groups. While consultations for anxiety/depression were consistently below the predicted levels, declines in ADHD occurred later in the pandemic. The data suggests an overall decline in healthcare use rather than a shift between sectors.

**Conclusion:**

The findings offer insights into healthcare use during pandemic times among children and adolescents with pre-existing mental health diagnoses. The study underscores the importance of continuous monitoring and support for this group, ensuring accessible and responsive healthcare during public health emergencies.

**Supplementary Information:**

The online version contains supplementary material available at 10.1186/s12888-024-06422-7.

## Introduction

The Covid-19 pandemic introduced complexities and challenges that were likely more demanding for some groups, including children and adolescents [1, 2]. These developmental stages are not only critical periods for the emergence of mental health conditions [3], but also times with fewer resources and experiences to draw upon to cope with the multiple challenges brought about by the pandemic, such as social isolation, disrupted routines, and familial stress [4]. This may have been particularly true for children and adolescents with pre-existing mental health conditions [5, 6]. Our study focuses on the long-term patterns of psychiatric healthcare use within this vulnerable group. The primary motivation is to gain insights into shifts in psychiatric healthcare use, which can lay the groundwork for future research aimed at identifying drivers of these changes in the context of a global public health crisis.

The literature on the COVID-19 pandemic’s effect on children’s and adolescents’ psychiatric healthcare use is marked by a lack of high-quality long-term studies beyond the initial lockdown phase and the first 18 months. At the onset of the pandemic, evidence primarily pointed to a reduction in mental healthcare contact [7–9]. As the pandemic progressed, several studies reported an uptick in psychiatric healthcare use [10–12]. For instance, a Canadian study showed that while physician-based outpatient weekly visits declined during the first months of the pandemic, this was followed by growth, to above expected levels, from summer 2020 and until the end of the study period in February 2021 [7]. A Danish Registry study, with data up until February 2021, also found an increase in children and adolescents’ psychiatric hospitalizations. However, these increases may also reflect a continuation of pre-pandemic trends of increased mental healthcare use among children and adolescents [13] rather than a response to the pandemic. Studies that separate changes due to the underlying societal trend of increased mental healthcare use from the unique stressors introduced by the pandemic can shed important light on the situation. A Norwegian study used a design that allowed for separating the shock of the pandemic from trends of increased mental healthcare use among children and adolescents [9]. The results showed a temporary reduction in mental health consultations during lockdown, followed by an increase in primary care volume during the fall of 2020 and the winter of 2021 and in specialist care from the spring of 2021 [9]. Considering these findings, it becomes evident that the interplay between the pandemic’s immediate impacts and the broader, pre-existing trends in mental healthcare use demands careful examination. The nuanced effects on specific subgroups remain insufficiently understood.

Certain characteristics among children and adolescents, such as pre-existing mental health conditions [5, 6, 14], may increase their vulnerability to mental health challenges and influence their likelihood of seeking help for such problems. It seems plausible that among those with pre-existing mental health conditions, symptoms may have worsened due to, for example, changes in routines and increased isolation, economic and health-related family stress, which could have reduced available emotional support. On the other hand, some may have experienced relief during the pandemic, such as reduced face-to-face bullying and less parental stress [15]. For instance, one study addressing mental health among a cohort of at-risk adolescents (determined by their self-reported emotional and behavioral symptoms in their first year of high school) before and during the pandemic showed a decrease in symptom scores during the pandemic [15]. Further, the pandemic appears to have impacted girls more significantly than boys [4, 6, 16, 17]. This may be due to their greater reliance on social connections [18, 19], which were disrupted during periods of social isolation. Additionally, girls’ tendency to express emotions and seek help may have amplified pre-existing gender disparities in mental healthcare use [20].

Healthcare use during the pandemic is likely to vary across different pre-existing psychiatric conditions, highlighting the importance of examining a range of these conditions within a single study [9, 21]. Such an approach allows for a more nuanced understanding of how the pandemic has influenced healthcare use, revealing potential differences in consultation patterns in both primary and specialist healthcare services.

A broad, generalized analysis of healthcare use risks overlooking important variations between groups with different conditions and leads to inaccurate conclusions. Detailed and nuanced examination of groups that are likely differently affected is therefore necessary, not only to capture the varied impacts of the pandemic on healthcare use but also to guide the development of targeted interventions and support strategies for those most affected. Further, systematic reviews on the mental health impact of COVID-19 among young individuals emphasize several research gaps, including a need for nationally or regionally representative studies, longitudinal cohort studies, and the need to factor in pre-existing psychiatric conditions [22].

To sum up, knowledge about the effects the pandemic has had on vulnerable children and adolescents’ mental healthcare use needs to be developed further; the current study contributes to this by examining psychiatric healthcare use among a particularly vulnerable group, namely children and adolescents with recent pre-existing mental health conditions, using a longitudinal cohort study, which includes all children and adolescents in Norway. The current study will contribute insight into the long-term impact of the pandemic on vulnerable children and adolescents’ mental healthcare use and how different subgroups may be differently affected during different phases of the pandemic. We use nationwide registry data, covering consultation with primary and specialist healthcare before and during the pandemic (2017–2022). Our first aim was to examine possible changes in mental healthcare consultation volume during the pandemic among boys and girls with any recent pre-existing mental health diagnosis or symptom code. We compare healthcare use during the pandemic with pre- pandemic patterns to assess any deviations from the baseline. Next, we estimate changes in the pattern of consultation volume within boys and girls with pre-existing diagnoses of the following conditions: (1) anxiety or depression, (2) Attention-Deficit Hyperactivity Disorders (ADHD). Results for (3) sleep problems, and (4) Autism Spectrum Diagnoses (ASD) are presented in the supplementary due to data available from only one registry for each condition.

## Methods

### Setting

From the early stages of the pandemic, Norway prioritized vulnerable groups. Healthcare services were instructed to maintain a focus on these populations, including children and adolescents, and individuals with mental health conditions. A dedicated project focusing on psychosocial aspects was established by the Norwegian Directorate of Health shortly after the onset of the pandemic. Regular meetings with user organizations were initiated to get continuous feedback on key issues regarding how the pandemic and the measures implemented to curb the spread of the pandemic influenced these groups, and expertise was pooled to support these groups. While some municipal personnel were reassigned to infection control tasks, additional funding was provided to ensure that this did not negatively impact individuals with mental health conditions. This was monitored through meetings with regional administrative leaders and officials overseeing the operations and governance of counties in Norway, who were responsible for ensuring that the government’s decisions, goals, and guidelines were followed.

### Context of mental health care for children and adolescents in Norway

In Norway, primary care, primarily provided by general practitioners (GPs), manages milder mental health conditions in children and adolescents and oversees medication management for conditions like ADHD. GPs also follow up on stable conditions after specialist services have established a diagnosis. For more severe or persistent conditions, GPs refer patients to specialist care. Specialist mental healthcare is offered through Child and Adolescent Mental Health Services (BUP), which handles more complex or severe conditions, often providing psychotherapy, behavioral interventions, and multidisciplinary evaluations.

### Data sources

#### Beredt C19

We used data from the Norwegian emergency preparedness register for COVID-19 [23], established at the Norwegian Institute of Public Health to learn about the pandemic’s spread and consequences in real-time. We used data from two key databases – The Norwegian Control and Payment of Health Reimbursement database (KUHR), from which we included data from 2017 to 2022, and The Norwegian Patient Registry (NPR), with data covering the period from 2017 to 2021. These were matched with demographic information from the National Population Registry data. Data from 2022 is presented in the supplementary materials.

We could not differentiate between in-person visits and telehealth consultations, as our dataset combined all types of healthcare contacts into a single measure. This aggregation, necessitated by data minimization strategies in the emergency preparedness register for this study, limits our ability to evaluate the potential impacts of the shift to telehealth during the pandemic.

#### Sample inclusion criteria

Our sample consists of all children and adolescents aged 6–19 years in Norway with recent (1 calendar year prior) pre-existing mental health conditions, defined as any registry record with codes indicating consultation with primary- and/or specialist healthcare services for *any* mental health symptom or diagnosis (Table 1).

#### Consultation volume in primary healthcare

We used data from KUHR to assess the frequency of weekly primary care consultations for mental health conditions among children and adolescents with recent pre-existing mental health diagnoses. Our analysis included all consultations for individuals aged 6–19 years registered with one or more of the diagnostic codes in Table 1. For each consultation, one or more diagnostic codes were registered and classified under the International Classification of Primary Care system, 2nd edition (ICPC-2) [24].

#### Consultation volume in specialist healthcare

We used data from NPR to examine the number of weekly consultations in specialist healthcare for mental health conditions among children and adolescents with pre-existing mental health diagnoses. Except in acute hospitalizations, specialist healthcare typically requires a general practitioner (GP) referral. Based on the International Classification of Diseases, 10th revision (ICD-10)26, health professionals registered one or more diagnostic codes for each consultation to describe the patient’s clinical problem. Diagnostic codes used in this analysis are listed in Table 1.

**Table 1:** Symptom and diagnostic codes included in analyses

	Primary healthcare	Specialist healthcare:
	International Classification of Primary Care (ICPC) codes: Psychological symptoms and disorders	International Classification of Diseases (ICD) codes: Mental disorders
*Any* mental health code	All Chapter P codes	All chapter F codes
Anxiety/depression	P01, P03, P74, P76, P79, P82	F32, F33, F40, F41, F43, F93.0, F93.1, F93.2
Sleep problems	P06	-
Attention-Deficit Hyperactivity Disorder (ADHD)	P81	F90
Autism Spectrum Diagnoses (ASD)	-	F84

Notes Names of the included ICPC codes: P01 Feeling anxious, P03 Feeling depressed, P74 Anxiety disorder, P76 Depressive disorder, P79 Phobia/obsessive-compulsive disorder, P81 ADHD, P82 Posttraumatic stress disorder, P06 Sleep problems. Names of the included ICD-10 codes: F32 Depressive episode, F33 Recurrent depressive disorder, F40 Phobic anxiety disorders, F41 Other anxiety disorders, F43 Reaction to severe stress and adjustment disorders, F93.0 Separation anxiety disorder, F93.1 Phobic anxiety disorder of childhood, F93.2 Social anxiety disorder of childhood. F90 Hyperkinetic disorders, F84 Autism spectrum diagnoses

### Ethics

The study was approved by the Norwegian Regional Committees for Medical and Health Research Ethics, approval number 2021/267,200.

### Design

**Fig. 1:**
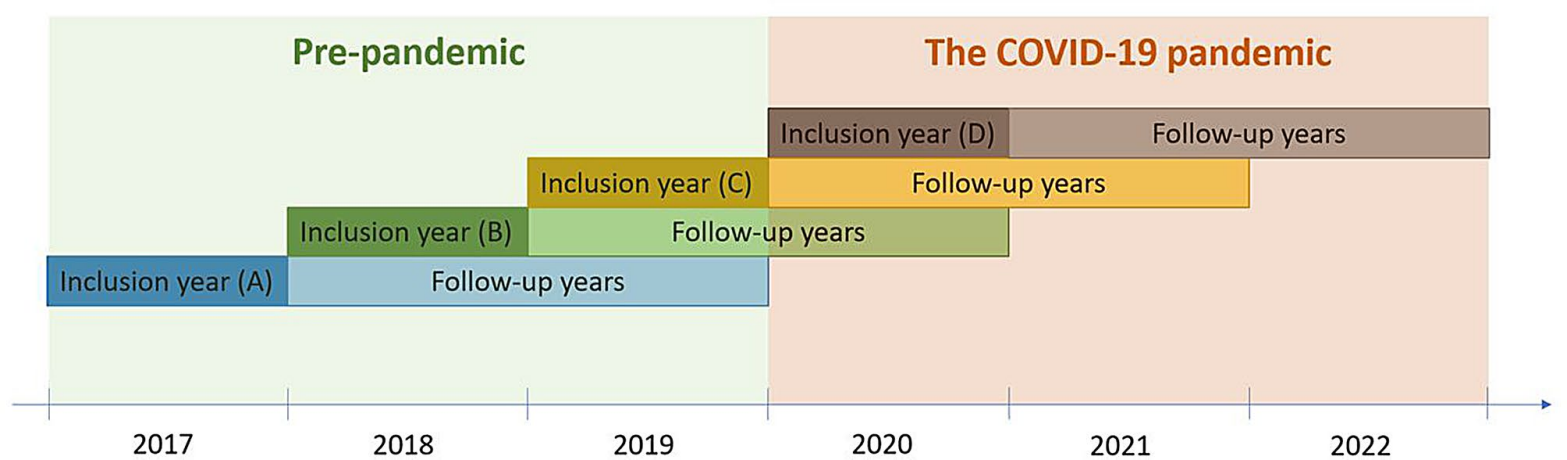
Illustration of the four datasets in the study. Inclusion year is the year we used to define individuals as having a pre-existing mental health diagnosis based on individuals having one or more weekly consultations in primary or specialist healthcare for one or more of the pre-selected symptoms and diagnoses

To compare the number of consultations for mental health conditions among individuals with pre-existing mental health diagnoses before and during the pandemic, we created four data sets (see Fig. 1 for details) based on four different inclusion years. The inclusion years define individuals as having a pre-existing condition, based on the pre-selected diagnoses and symptom codes outlined above.

The pre-pandemic dataset (A) includes data from individuals diagnosed with one or more of the pre-selected mental health conditions in 2017, followed through 2018 and 2019. This dataset is used to make predictions for datasets B, C, and D. We assume that the variability observed from 2017 to 2019 represents any typical three-year period. A semi-pre-pandemic dataset (B) comprises data from individuals identified with a mental health diagnosis in 2018 and followed up through 2019 and 2020. Further, a pandemic dataset (C) included individuals identified in 2019, with follow-up data during the pandemic in 2020 and 2021. We also included a pandemic dataset for primary healthcare only (D), focusing on individuals first identified in 2020, with follow-up extending into 2021 and 2022.

It is important to note that an individual could have had multiple consultations within a single week. However, in our data sets, an individual is only counted once during a respective week. Furthermore, there is a possibility of overlap in consultation data between these datasets, particularly for 2019 and 2020, where an individual’s consultations might be represented in three data sets.

In this study, strict social distancing refers to periods when the Norwegian government implemented national regulations to limit social contact. These measures included restrictions on public gatherings, periods with home school, limitations on social interactions outside of households, and recommendations for remote work.

### Statistical analyses

All statistical analyses and data visualizations in this study were conducted using R Studio Build 386 2023.03.0 [25, 26]. For simulations, we utilized the ‘boot’ package [27], while ‘ggplot2’ [28] was employed for visualizing the time series data. Initially, we aggregated the total number of weekly consultations per 100.000 individuals. Dataset A corresponds to the pre-pandemic era. Given that our predictive analysis was based on only one pre-pandemic period, we bootstrapped the Pearson correlation coefficient to estimate the mean correlation. This estimation involved assessing the correlation between weekly consultations in 2017 (the year of inclusion in data set A) and each subsequent follow-up year (2018 and 2019). This process was achieved through 10,000 bootstrap samples, randomly drawn with replacement from Dataset A. In our model, these mean Pearson correlation coefficients were treated as constant values. However, in the bootstrap samples, the weekly consultation numbers varied. Consequently, the mean Pearson correlation coefficient was calculated from 10,000 distinct data combinations, providing a robust estimation under varying scenarios.

To compare the consultations rates between primary and specialist healthcare across different years, we performed a paired t-test. The test assessed whether there was a statistically significant difference in mean consultation rates per 100.000 individuals between the two groups. Each pair represented the same year for primary and specialist consultations, making this test suitable for detecting differences in consultation patterns over time. Assumptions of the paired t-test, such as the normality of the differences, were checked, and a 95% confidence interval was calculated for the mean difference to quantify the range of plausible values for the true difference.

In our model, we assume that the variability observed from 2017 to 2019 is representative of any typical three-year period. Therefore, by drawing 10,000 random samples from this data, we simulated a distribution of correlation coefficients across different years. This simulation enables us to infer potential outcomes in subsequent years, assuming the pandemic had no impact on consultation rates. To forecast the 2020 consultation rate per 100,000 for the 2019 cohort (dataset C), we multiplied the observed weekly consultation rate in 2019 (pandemic inclusion year) by the mean Pearson correlation coefficient calculated between 2017 and 2018 from the pre-pandemic data set (A). We replicated this method to project 2021’s weekly consultation rate, using the mean correlation coefficient between 2017 and 2019. For these predictions, we employed Loess Regression with a 99.9% Confidence Interval (CI) to contrast the predicted and observed weekly consultation rates in 2020 and 2021 from the pandemic cohort (dataset C). A 99.9% CI was chosen due to our reliance on a single pre-pandemic observation period for predictions. In our time series plots, both for predicted and observed data, we applied a smoothed line. The degree of smoothing was determined for each analysis to clearly depict the overall trend using the ‘geom_smooth()’ function in R’s ggplot2 package.

To quantitatively assess differences in trends between sex categories and between the predicted and observed primary healthcare consultations for 6-19-year-old patients, linear regression models were used for the periods 2019–2020 and 2020–2021. The models included time, sex, and type (observed vs. predicted) as factors, along with their interactions. Time was represented as the number of weeks from the start of each period, and sex was treated as a categorical variable. The consultation data were reshaped into long format, allowing for the comparison between observed and predicted values over time for both boys and girls. The models were fitted using the “lm” function in R, with the response variable being the weekly consultation rate per 100.000 individuals.

The main effects and interactions were examined to identify significant differences. For each period, the intercept represented the baseline for boys’ observed values at the start of the period. The models assessed whether there were statistically significant differences in the consultation rates based on sex and type, and if trends over time varied between observed and predicted values. Interaction terms were included to detect any potential differences in trends over time based on the combined effects of time, sex and type. Significant levels for each term were evaluated using p-values, and model fit was assessed using the R-squared value and overall F-test.

All models were stratified by sex. To streamline the results and ensure consistency, we have limited the main results section to findings for which we have data from both databases (KUHR and NPR). Thus, for clarity, we have focused the results section on diagnostic groups covered in both databases, specifically anxiety/depression and ADHD. Results for diagnostic/symptom codes only covered in one registry, namely ASD and sleep problems, are provided in the supplementary material to ensure the main findings remain clear and accessible, while still allowing readers access to the full range of data. Further, as we only have primary care data for 2022, those results are included in the supplementary material as sFigures 1–3, allowing us to maintain focus on the key comparisons across both healthcare sectors while still providing the full range of data for interested readers.

## Results

See Table 2 for descriptive statistics for mean mental health consultations for children and adolescents [6–19] in primary and specialist healthcare for any mental health consultation per week and rate per 100 000.

**Table 2:** Descriptive statistics of any mental health consultations for children and adolescents (ages 6–19) in primary and specialist healthcare, reflecting cohort overlap

		Primary healthcare: Any mental health consultation		Specialist healthcare: Any mental health consultation	
Dataset	Year	Mean consultations per week (*N*)	Mean consultation rate per 100 000	Mean consultations per week (*N*)	Mean consultation rate per 100 000
**A**: Pre-pandemic					
Inclusion year	2017	2392	267	8604	960
First follow-up year	2018	1302	145	5897	656
Second follow-up year	2019	1197	133	4082	454
**B** Semi-pre-pandemic					
Inclusion year	2018	2454	273	8183	911
First follow-up year	2019	1339	149	5987	666
Second follow-up year	2020	1247	139	4152	463
**C** Pandemic 1					
Inclusion year	2019	2492	277	8494	944
First follow-up year	2020	1378	154	6318	704
Second follow-up year	2021	1448	162	2883	322
**D** Pandemic 2					
Inclusion year	2020	2608	291	-	-
First follow-up year	2021	1656	185	-	-
Second follow-up year	2022	1378	154	-	-

Note The variability observed in the consultation rates across the same calendar year is due to differences in the datasets, which have distinct inclusion criteria, follow-up periods, and patient cohorts. Some overlap occurs as some individuals are followed across multiple time frames, leading to discrepancies in consultation rates. These differences should be considered when interpreting the data

A paired t-test was conducted to compare the mean consultation rates per 100.000 between primary healthcare and specialist healthcare across the three time periods: pre-pandemic (2017–2019), semi-pre-pandemic (2018–2020), and pandemic (2019–2021). The analysis included a total of 9 time points with corresponding data from both healthcare settings.

The mean consultation rate per 100.000 was significantly lower in primary healthcare (M = 188.78) compared to specialist healthcare (M = 675.56), with an average difference of -486.78 consultations per 100.000 (95% CI [-626.45, -347.11]). The paired t-test revealed a statistically significant difference between the two groups, t (8) = -8.04, *p* < 0.001, indicating that specialist healthcare consistently had a higher mean consultation rate per 100.000 than primary healthcare during the studied periods.

### Mental health contacts overall

**Fig. 2:**
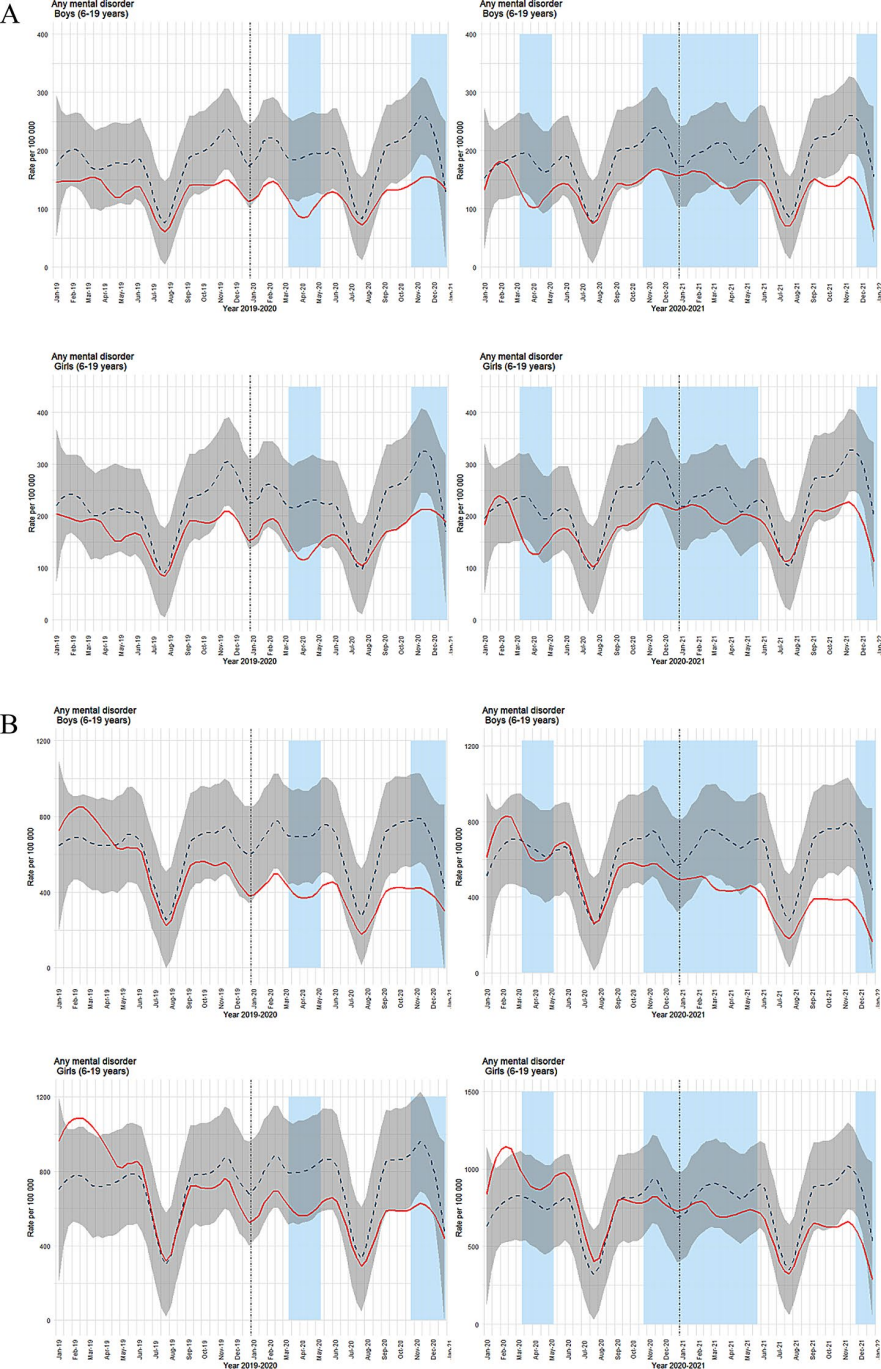
(**a**) Time series plots for weekly consultations in primary healthcare (ICPC-2 codes, chapter P). (**b**) Time series plots for weekly consultations in specialist healthcare (ICD codes, chapter F). Both figures show consultations per 100,000 (solid red line) for boys (top row) and girls (bottom row). The dashed blue line represents predicted consultations with a 99.9% confidence interval (grey shading). Light blue columns indicate periods of strict social distancing measures by the Norwegian government. The left column shows results for inclusion year 2018 with follow-up years 2019–2020, while the right column shows inclusion year 2019 with follow-up years 2020–21

In 2019, weekly primary healthcare contacts for any mental health condition in children and adolescents with registered mental health diagnoses in 2018 (data set B) largely met the predicted levels, aside from a reduction in November and December (Fig. 2a). In 2020, boys’ contacts declined around the first and second social restriction phases, with similar patterns for girls but to a lesser extent. For those registered with mental health diagnoses in 2019 (data set C), boys experienced a drop in March and April 2020 during the initial social distancing, with a slight reduction in March 2021 and a more pronounced drop from September to year-end (Fig. 2a). Girls’ consultations fell in March and April 2020 but stayed within predicted ranges until October 2021, when there was a drop that lasted through the end of the year. Boys registered with mental health diagnoses in 2020 (data set D) began 2021 with the weekly number of consultations as anticipated. However, they dropped from October 2021 to February 2022, aligning with social restrictions, and then again from April to June and August to December (Fig. 2a). Girls had slightly higher than predicted consultations at the beginning of 2021, followed by several periods of reductions from October 2021. The overarching trend shows a more notable drop in consultations among boys throughout the pandemic, with lower-than-predicted weekly consultations becoming increasingly apparent over time. In all data sets, boys consistently showed longer and more pronounced periods of reduced consultation than girls.

In specialist healthcare, boys with mental health diagnoses registered in 2018 (data set B) showed a clear drop in weekly consultations from February to June 2020, encompassing the initial phase of strict social distancing (Fig. 2b). A subsequent drop was noted from September to December, before, during, and immediately after the second social distancing phase. For girls, while early 2019 saw higher than predicted consultations, the trend generally matched the predicted levels until the end of 2020, when a drop corresponded with the second social distancing phase. For those recorded with mental health diagnoses in 2019 (data set C), boys’ consultations in 2020 remained as predicted until a drop from February to June 2021, aligning with a phase of social restrictions, followed by another period with lower-than-predicted consultation from August 2021 through year-end (Fig. 2b). Girls had slightly higher-than-predicted consultations in early 2020. However, for most of the follow-up period, observed consultations matched the predicted levels, except for a drop at the end of 2021, coinciding with a new phase of social restrictions.

To quantitatively support the visual differences in trends between sex groups and between predicted and observed primary healthcare consultations for 6–19-year-old patients per week, we conducted linear regression analyses for the 2019–2020 and 2020–2021 periods. The models included time, sex, and type (observed versus predicted) as factors, along with their interactions. The results are shown in Table 3.

The 2020–2021 model intercept was higher than that for 2019–2020, indicating an overall increase in baseline values. This suggests that the starting level for boys’ observed values was higher in 2020–2021 than in 2019–2020, possibly due to changes in external factors influencing the data.

In the 2019–2020 period, a significant difference was observed between predicted and observed values (*p* = 0.008), with predicted values generally higher than observed values. However, this difference was not statistically significant in the 2020–2021 period (*p* = 0.121), indicating that the gap between the predicted and observed values narrowed. Girls consistently had higher consultation rates than boys in both periods, with statistically significant differences (*p* = 0.010) for 2019–2020 and (*p* = 0.022) for 2020–2021. Although the magnitude of this difference slightly decreased in the 2020–2021 period, the effect remained significant.

The interaction between time and type (observed versus predicted) was significant in 2020–2021 (*p* = 0.033), indicating that the trend over time differed between observed and predicted values during this period. In contrast, this interaction was not significant in 2019–2020 (*p* = 0.104), suggesting that the relationship between observed and predicted values was more stable in the earlier period. We did not find significant interactions involving sex and time in either period (*p* > 0.1), indicating no strong evidence that the trends over time varied simultaneously by sex and type.

**Table 3:** Estimates of weekly consultations in primary healthcare for patients aged 6–19 years. Effects of time, consultation type, and sex (2019–2020 and 2020–2021)

Period	Term	Estimate	Std.Error	t-value	*p*-value
2019–2020	(Intercept)	129.83	10.27	12.64	< 0,001
	Time	-0.09	0.17	-0.53	0.597
	Type (Predicted)	38.61	14.52	2.66	0.008
	Sex (Girls)	37.64	14.52	2.59	0.010
	Time × Type (Predicted)	0.39	0.24	1.63	0.104
	Time × Sex (Girls)	0.07	0.24	0.29	0.770
	Type (Predicted) × Sex (Girls)	-4.68	20.54	-0.23	0.820
	Time × Type × Sex	0.03	0.34	0.09	0.926
2020–2021	(Intercept)	141.45	10.39	13.62	< 0,001
	Time	-0.11	0.17	-0.63	0.531
	Type (Predicted)	22.84	14.69	1.56	0.121
	Sex (Girls)	33.85	14.69	2.30	0.022
	Time × Type (Predicted)	0.52	0.24	2.14	0.033
	Time × Sex (Girls)	0.27	0.24	12.24	0.266
	Type (Predicted) × Sex (Girls)	2.15	20.77	0.10	0.918
	Time × Type × Sex	-0.17	0.34	-0.51	0.612

Overall, the results indicate more extended periods of reduced specialist healthcare contacts for boys than for girls and that the reductions were more pronounced. This contrasted with primary care, where boys and girls experienced about the same periods and levels with reduced contact. Next, we focus on diagnostic groups, specifically depression/anxiety, and ADHD, for which we have data from both primary and specialist healthcare. Results for sleep problems, available only from primary care, and ASD, available only from specialist healthcare, are presented in the supplement as sFigures 4 and 5.

### Anxiety/depression

**Fig. 3:**
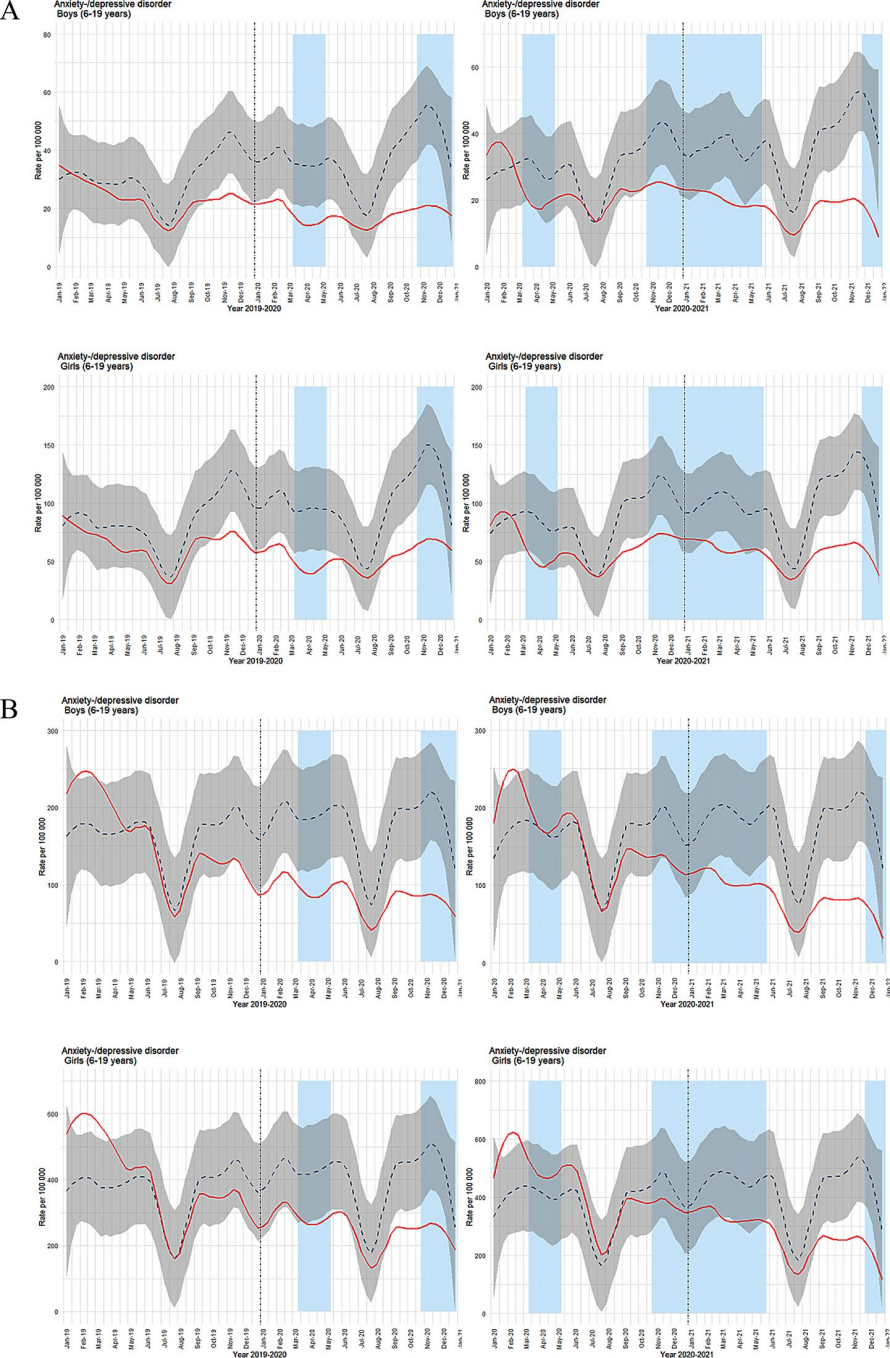
**a** Time series plots for observed weekly consultations in primary healthcare for anxiety/depression (ICPC-2 codes: P01, P03, P74, P76, P79, P81, P82). **b** Time series plots for observed weekly consultations in specialist healthcare for anxiety/depression (ICD codes: F32, F33, F40, F41, F43, F93.0, F93.1, F93.2). For both figures, observed consultations per 100,000 are shown as a solid red line, with predicted consultations represented by a dashed blue line and 99.9% confidence interval in grey shading. Light blue columns indicate periods of strict social distancing measures implemented by the Norwegian government. The left column displays results for inclusion year 2018 with follow-up years 2019–2020, while the right column shows inclusion year 2019 with follow-up years 2020–2021. Results for boys are in the top row, and for girls in the bottom row

In primary healthcare for anxiety/depression, we observed periods of lower-than-predicted weekly contacts throughout the observation periods. (Fig. 3a). The pattern was quite similar for boys and girls, and reductions were not limited to social restriction phases. Specialist healthcare showed a different trend (Fig. 3b): periods with lower-than-predicted weekly contacts were both longer and more marked for boys than girls, deviating from the relatively equal duration and degree of contact reduction seen in primary care.

### ADHD

**Fig. 4:**
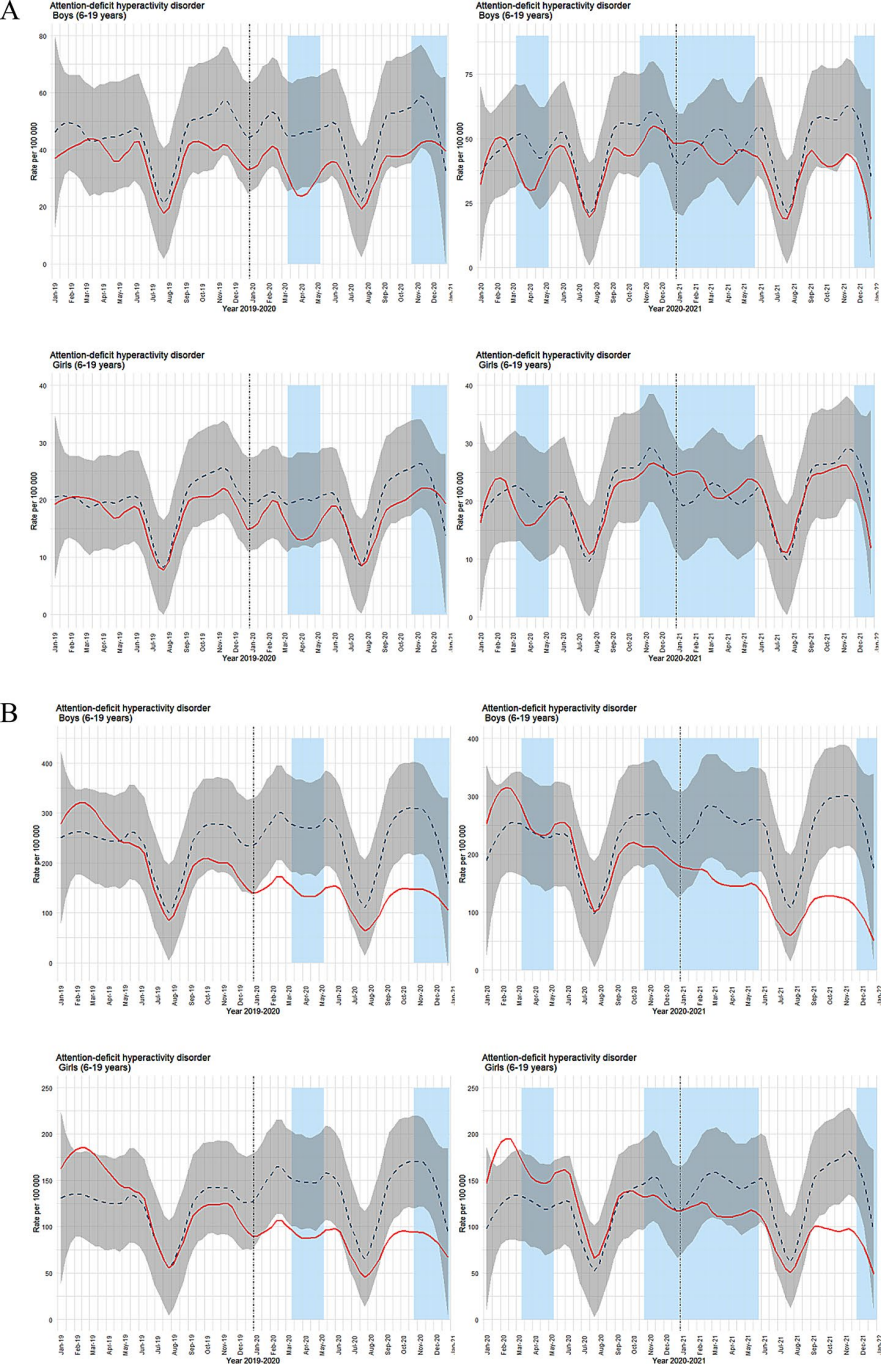
(**a**) Time series plots for weekly consultations for ADHD in primary healthcare (ICPC-2 code: P81). (**b**) Time series plots for weekly consultations for ADHD in specialist healthcare (ICD code: F90). For both figures, observed consultations per 100,000 are shown as a solid red line, with predicted consultations represented by a dashed blue line and 99.9% confidence interval in grey shading. Light blue columns indicate periods of strict social distancing measures implemented by the Norwegian government. The left column displays results for inclusion year 2018 with follow-up years 2019–2020, while the right column shows inclusion year 2019 with follow-up years 2020–2021. Results for boys are in the top row, and for girls in the bottom row

In primary healthcare, observed weekly ADHD contacts for both boys and girls were largely in line with the predicted levels in the initial periods (Fig. 4a). The exception was a minor reduction for boys in the first restriction phase in March and April 2020. As the pandemic progressed, deviations emerged for both genders, with boys experiencing more prolonged periods of reduced contact. In specialist healthcare, lower-than-predicted weekly contacts were noted from the beginning of 2020 (Fig. 4b). Consistent with primary care, boys showed longer durations of reduced ADHD contacts. However, the reductions were more pronounced in the specialist setting compared to primary healthcare.

## Discussion

### Main findings

We examined if there were changes in weekly consultation volume for psychiatric symptoms and conditions among children and adolescents with pre-existing mental health diagnoses across different time-periods during the pandemic, compared to a pre-pandemic period. There was a consistent trend of periods with lower-than-predicted weekly consultations for mental health overall in both primary and specialist healthcare, and this became more pronounced in later follow-up periods. The consistent reduction in weekly consultation volumes across healthcare settings, compared to pre-pandemic levels — and becoming more pronounced over time — suggests a genuine decline in mental healthcare use. Further, trends varied across healthcare services, across diagnostic groups, and between boys and girls. The number of weekly contacts for mental health overall in primary healthcare showed trends of a notable decline in contacts for boys, with more extended and more pronounced periods of reduced contact as the pandemic progressed. Girls showed a similar but less pronounced pattern. Specialist healthcare data mirrored these findings, with the trend of boys having more extended periods of reduced number of weekly consultations and more pronounced reductions than girls. The differences in weekly consultation volumes between boys and girls were more pronounced in specialist healthcare.

Our study also examined healthcare use for specific diagnostic groups. The patterns of observed versus predicted consultation volumes varied across diagnostic groups, revealing the nuanced ways the pandemic (directly or indirectly) affected children and adolescents with different mental health conditions. Lower-than-predicted consultations were particularly long-lasting for some diagnostic groups. For instance, weekly consultations for anxiety/depression in primary healthcare were lower than predicted throughout much of the observation periods for both boys and girls, but with trends of more pronounced reductions in later follow-up periods.

A notable finding is the trend of gender disparity in healthcare consultation volume for different diagnostic categories. For instance, in specialist healthcare, boys faced longer and more significant reductions in contacts for anxiety/depression compared to girls, diverging from the relatively equal patterns seen in primary care. For other conditions, like ADHD, lower-than-predicted weekly consultation volumes in primary healthcare occur later into the follow-up period for both boys and girls, with trends of longer durations of lower-than-predicted consultation among boys. This pattern differs from consultations for ADHD in specialist healthcare, where both boys and girls have periods with lower-than-predicted consultation starting just prior to the pandemic. Our analyses identify disparities in mental healthcare use during the pandemic, but do not shed light on the underlying mechanisms driving these trends.

Our findings of reduced consultation volumes for mental health during the pandemic contrast with a Norwegian study by Evensen and colleagues, who also used registry data on psychiatric healthcare use for Norwegian children and adolescents [9]. That study, which covered *all* children and adolescents with mental health consultations, showed that after temporary reductions during the initial lockdown, there was an overall increase in consultations [9]. They also examined specific conditions that we addressed, anxiety/depression and ADHD, and observed an increase in these conditions [9]. Our study is the first to address psychiatric healthcare use during the pandemic among children and adolescents with pre-existing mental health conditions, so there is limited existing research for comparison. A Norwegian study on adults with pre-existing mental health conditions found an increase in primary healthcare consultations during the pandemic, contrasting with our findings in children and adolescents [21]. Both studies used registry data and a similar design. However, the adult study followed a shorter period and used only two time periods: pre-pandemic and pandemic [21]. Likewise, while our study aligns with Evensen et al.‘s study [9] in terms of health registry data for children and adolescents, key differences exist: their broader focus on all young people in contact with mental healthcare services and the use of a difference-in-difference design. These differences should be taken into consideration when comparing the results. A Norwegian survey-based study assessing the long-term impact of the Covid-19 pandemic on the mental health symptoms of children and adolescents, found that children with pre-existing problems self-reported more mental health symptoms during the pandemic compared to their peers [14]. Considering our findings alongside these studies, one interpretation is that individuals most impacted by the pandemic had reduced healthcare consultations.

The reasons for the decline in consultations remain uncertain. One possibility is that those with pre-existing conditions and their parents were more worried about COVID-19 and kept their distance, thus affecting help-seeking behavior. The decrease in consultations may partly result from reduced monitoring by teachers during the pandemic, as they often detect changes in children with pre-existing diagnoses that caregivers may overlook. Another possibility is that some young individuals with pre-existing mental health diagnoses felt less alone during the pandemic due to shared experiences of struggling with social restrictions, offering some relief. User organizations’ data support this, as they saw a reduction in “recurring contacts” and an increase in “new contacts”. This suggests that the pandemic influenced help-seeking patterns. However, this explanation seems more plausible for some conditions than others. For instance, those with anxiety-related conditions may have felt relief from reduced social interaction, temporarily lowering their need for healthcare [29]. In contrast, individuals with ADHD who rely on structured environments may have experienced worsened symptoms [30]. However, reduced demands, such as fewer in-person lectures and less homework, may have contributed to the decrease in consultations, as some experienced symptom relief due to fewer settings requiring sustained attention.

Our findings highlight the importance of understanding how the pandemic impacted different groups/conditions. A key question is which groups contributed to the increase observed in the study by Evensen and colleagues [9], if not those with pre-existing mental health conditions. About 11% of young individuals who responded to Youth Mental Health Norway’s survey reported experiencing mental health challenges for the first time during the pandemic [31]. This suggests that the increase observed by Evensen may reflect new patients, potentially straining healthcare services [9]. However, this would likely have resulted in a shift towards primary healthcare, which is more flexible in handling increased demand. However, our observations do not suggest such a shift. Additionally, youth with pre-existing conditions like ADHD, who were already stabilized on long-term medication, may have required fewer follow-ups, providing a possible alternative explanation for the differing findings. Further, if an individual’s symptoms (and diagnosis) indicate a right to specialist healthcare, a shift back to primary care is unlikely. An exception might be ADHD, which is often followed up by general practitioners. The different changes in ADHD consultation patterns between primary and specialist care may reflect the nature of ADHD treatment and the restructuring of healthcare services during the pandemic. In primary care, where ADHD management often involves routine medication monitoring, the observed trends were largely consistent with predicted levels, with relatively minor disruptions. In contrast, specialist care, which treats more complex cases, experienced significant disruptions from the onset of the pandemic. Teletherapy was available during the pandemic, but some families may have preferred in-person sessions, contributing to the decrease in consultations. The findings highlight the need for in-depth research to understand how the pandemic influenced healthcare prioritization and its impact on different patient groups. Our findings may contribute to reevaluating existing healthcare use models, especially in crises such as pandemics. It could inform revised theoretical frameworks that better explain how major stressors, like the pandemic, influence healthcare-seeking behavior across different groups. In light of the allostatic load theory [32, 33], we would expect that the heightened stress due to the pandemic would result in an increased demand for mental health services among vulnerable children and adolescents. While this was observed in previous studies focusing on the adult population of individuals with pre-existing mental health challenges [21] and in children and adolescents overall [9], we observe the opposite in children and adolescents with pre-existing mental health conditions. The findings could inform refinements or expansions of the theory to account for the observed boundary condition. For instance, some may avoid seeking help because they think they cannot get help during the pandemic. In times of crisis, the family’s role magnifies - for better or worse. For example, while mental health conditions are more prevalent in families with lower socioeconomic (SES) backgrounds [34], it is plausible that these families were less likely to seek or advocate for healthcare services under the high-pressure conditions of the pandemic compared to families with higher SES [35].

The different patterns observed among boys and girls may be due to a general tendency for less help-seeking behavior among boys; this is also reflected in the prevalence of girls versus boys who reach out to Youth Mental Health Norway for support. This tendency could have been exacerbated during the pandemic due to social isolation and the removal of structured environments like schools [36], which can play a critical role in addressing mental health conditions. Further examination of help-seeking behavior and the differences between boys and girls in this context is important.

The findings also open new avenues for research to explore why children and adolescents with pre-existing psychiatric diagnoses did not follow the expected trend of increased healthcare consultation during the pandemic. Our study does not capture the need for psychiatric healthcare, only actual contact. Some children and adolescents – or their parents – may have delayed seeking healthcare due to fear of infection and the tendency to stay home during periods of social restrictions. However, while periods with reduced mental healthcare use included periods with social restrictions, reductions in consultation occurred outside these periods too. The findings may also indicate a gap in mental healthcare service provision to this group of children and adolescents during crises. If so, understanding what these barriers are is essential for developing targeted interventions to improve healthcare access for vulnerable groups during future crises. Given these observations, monitoring trends in healthcare consultations for these children and adolescents post-pandemic becomes crucial to understand if and when they return to seek help in the healthcare system.

Healthcare systems may need targeted resource allocation during crises. Future health crisis management plans should consider strategies explicitly designed to support psychiatric healthcare services beyond general instructions to prioritize vulnerable groups or allocate additional funding. Given the observed declines in healthcare consultations during pandemics for certain groups, one potential approach could involve outreach programs targeting those most in need, such as children and adolescents with pre-existing mental health conditions, particularly boys.

### Methodological considerations

There are several strengths of this study. It builds on previous research that primarily relied on self-report data to examine mental health changes during the pandemic In contrast, we focused on actual contact with psychiatric healthcare services. Using longitudinal registry data, with minimal attrition provides a robust basis for studying healthcare use over time. The comprehensive coverage of these registries enhances the generalizability of our results, particularly in contexts with similar healthcare systems and pandemic-related restrictions. Additionally, using registry data mirrors real-world clinical practice, strengthening the external validity of our findings and offering valuable insights into actual healthcare use patterns. Further, utilizing both primary and specialist healthcare registries is a strength: understanding how the pandemic impacted different mental health service use levels can contribute to better preparation and resource allocation for future crises, ensuring more resilient and responsive mental healthcare systems.

However, our study also has several limitations. We had access to data from 2017 to 2022, which limited us to only one pre-pandemic observation period before the COVID-19 outbreak (2017–2019). To address this limitation and better estimate the correlation coefficients for making predictions, we employed a bootstrap method to offset the constraints posed by the restricted time frame of data availability. In our datasets, individuals were classified as having a pre-existing mental health condition based on data from one inclusion year. We do not know whether these identified patients had regular contact with healthcare services for mental health conditions before the years used for inclusion. If we had a more extended time series with inclusion years and follow-up years going back in time, we would have better represented the natural variation for the phenomenon we are studying. Further, our model assumed that the variation in 2017–2019 represented the variation in any given three-year period, which may not be the case. By relying on registry data, we capture only a subset of children and adolescents with mental health conditions, namely those in contact with healthcare services. Thus, the many children with such conditions who are not in treatment are not included. Further, while registry data are extensive, they lack crucial details such as information about symptom severity or quality of life. Our focus on predefined quantitative data overshadows the qualitative aspects of mental healthcare use and patient experiences. Additionally, the accuracy and completeness of registry data can be compromised by inconsistencies in data entry, which can impact the overall data quality. In this study, we analyzed young individuals (ages 6–19) as a single group, but we acknowledge that the prevalence and expression of psychiatric diagnoses can vary significantly across age groups. While age stratification was beyond the scope of this study, future studies should prioritize this approach to provide more nuanced age-specific insights. Further, we could not distinguish between in-person visits and telehealth consultations, as our dataset combined all types of healthcare contacts into a single measure. Due to data minimization strategies in the emergency preparedness register, this data aggregation limits our ability to assess the potential impacts of the shift to telehealth during the pandemic. Additionally, it is important to note that we used a single ICD code to capture “preexisting diagnosis”, and that this may not confirm a definitive diagnosis but could reflect a suspicion or the need for further follow-up to clarify.

## Conclusion

Our study, examining psychiatric healthcare use among Norwegian children and adolescents with pre-existing mental health conditions, shows a trend of lower-than-expected weekly healthcare consultations across primary and specialist healthcare during the pandemic. The findings highlight the nuanced impact of the pandemic on psychiatric healthcare use, including disparities across diagnostic groups, genders, and types of services. Notably, gender differences stand out, with boys experiencing more pronounced reductions in consultations.

## Electronic supplementary material

Below is the link to the electronic supplementary material.

Supplementary Material 1

## Data Availability

The data used in this study contains information from public health registries as part of Beredt C19, which is not made publicly available due to legal restrictions. However, researchers may request linked data from the same health registries used in this study by filling out an electronic application at Helsedata.no (https://helsedata.no/en/).

## Abbreviations

ADHD: Attention deficit hyperactivity disorder
ASD: Autism spectrum disorder
Beredt C19: Norwegian emergency preparedness register for COVID-19
CI: Confidence Interval
COVID-19: Coronavirus disease 2019
ICD-10: International classification of diseases, tenth revision
ICPC-2: International Classification of Primary Care system, 2nd edition
KUHR: The Norwegian Control and Payment of Health Reimbursements Database
SES: Socioeconomic status
