# Supplementary material for: Patterns of psychiatric healthcare use during pandemic times among boys and girls with pre-existing diagnoses: a Norwegian nationwide primary and specialist healthcare registry study
Source: BMC Psychiatry. 2025 Jan 22;25:68. doi: 10.1186/s12888-024-06422-7 (PMC11756222; doi:10.1186/s12888-024-06422-7)
Supplement: Supplementary file 1 — Supplementary Material 1 [file 12888_2024_6422_MOESM1_ESM.docx]

Patterns of psychiatric healthcare use during pandemic times among boys and girls with pre-existing diagnoses: A Norwegian nationwide primary and specialist healthcare registry study - Supplementary information

Contents

[**sTable 1**. Descriptive statistics of primary healthcare consultations by sex and diagnosis for 6–19-year-olds. 2](#_Toc184997224)

[**sFigure 1**: All mental health contacts in primary care for boys and girls (ages 6–19) including 2022 4](#_Toc184997225)

[**sFigure2**: Anxiety/depression contacts in primary care for boys and girls (ages 6–19) – including 2022 5](#_Toc184997226)

[**sFigure3:** ADHD contacts in primary care for boys and girls (ages 6–19) – including 2022 6](#_Toc184997227)

[**sFigure 4**: Sleep problems in primary care for boys and girls (ages 6–19) 7](#_Toc184997228)

[**sFigure 5**: Autism spectrum diagnoses in specialist care for boys and girls (ages 6–19) 9](#_Toc184997229)

Descriptive statistics for the population are presented in sTable 1. The consultations in the primary healthcare are stratified by sex and diagnosis. The number of inclusions for all mental health consultations increased over the period of 2017-2020 for both boys and girls. The number of inclusions were also increasing for consultations due to anxiety and depression, but less so for ADHD

### **sTable 1**. Descriptive statistics of primary healthcare consultations by sex and diagnosis for 6–19-year-olds.

| **Characteristics** | **Included 2017** | | | **Included 2018** | | | **Included 2019** | | | | **Included 2020** | | |
| --- | --- | --- | --- | --- | --- | --- | --- | --- | --- | --- | --- | --- | --- |
|  | Total | Males | Females | Total | Males | Females | Total | Males | Females | Total | | Males | Females |
| **Total population** (6-19 years) | 895958 | 460330 | 435628 | 898292 | 461622 | 436670 | 899318 | 462067 | 437251 | 897590 | | 460510 | 437080 |
| **Consultations** (6-19 years) |  |  |  |  |  |  |  |  |  |  | |  |  |
| Any mental health | 124389 | 59275 | 65114 | 127583 | 61136 | 66447 | 129571 | 61729 | 67842 | 138217 | | 65115 | 73102 |
| Anxiety/depression | 39412 | 12290 | 27122 | 41924 | 13819 | 28105 | 42011 | 13515 | 28496 | 42596 | | 13327 | 29269 |
| ADHD | 22503 | 15595 | 6908 | 22097 | 15271 | 6826 | 23261 | 15898 | 7363 | 26882 | | 18065 | 8817 |

Supplementary Figures 1-3 correspond directly to Figures 2a, 3a, and 4a from the main results section. They illustrate the same data on primary care contact for mental health conditions overall, for anxiety/depression and ADHD. However, the supplementary figures include an additional column displaying data from 2022, providing an extended view of the trends beyond what is presented in the main figures.

### **sFigure 1**: All mental health contacts in primary care for boys and girls (ages 6–19) including 2022


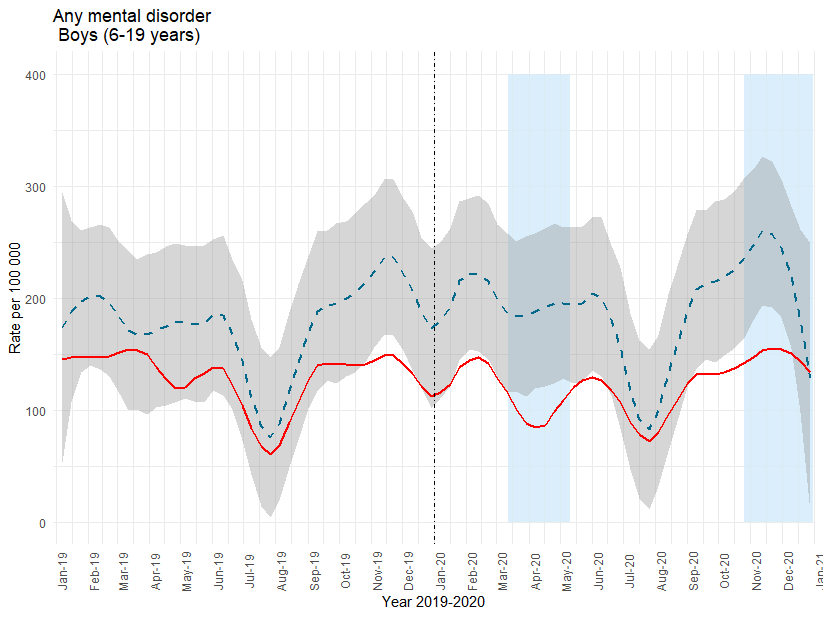

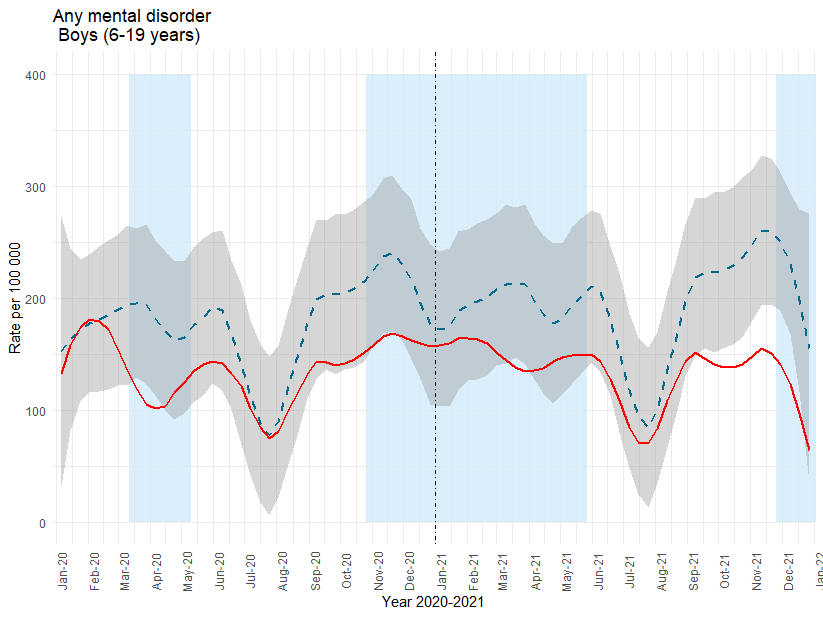

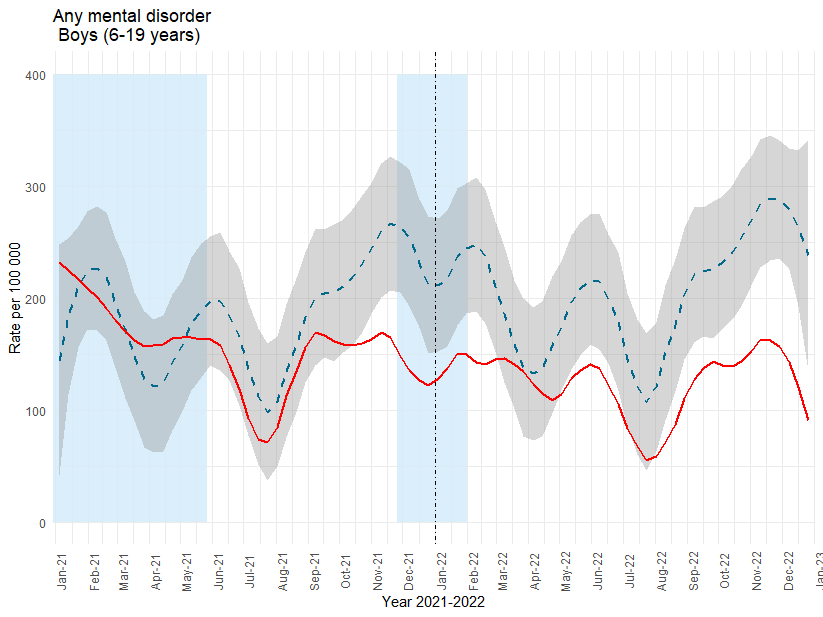


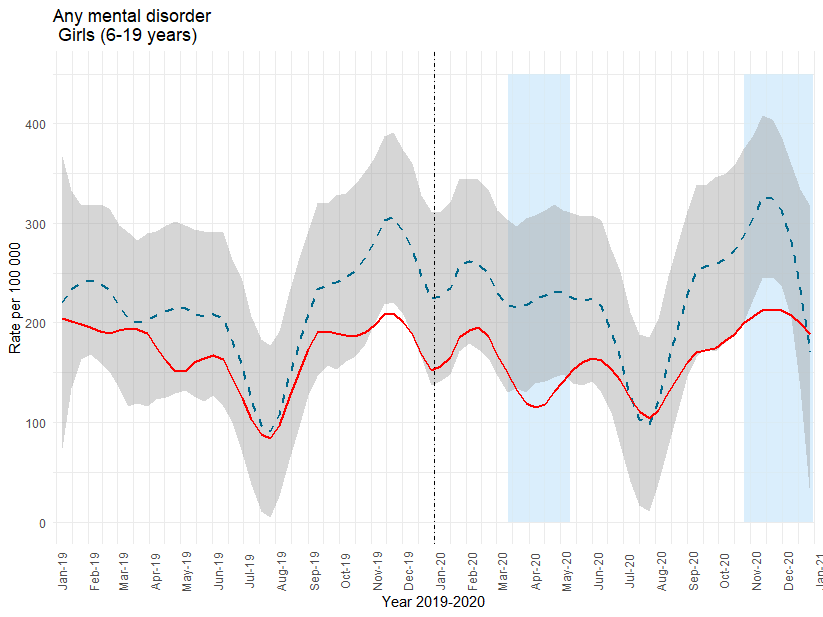

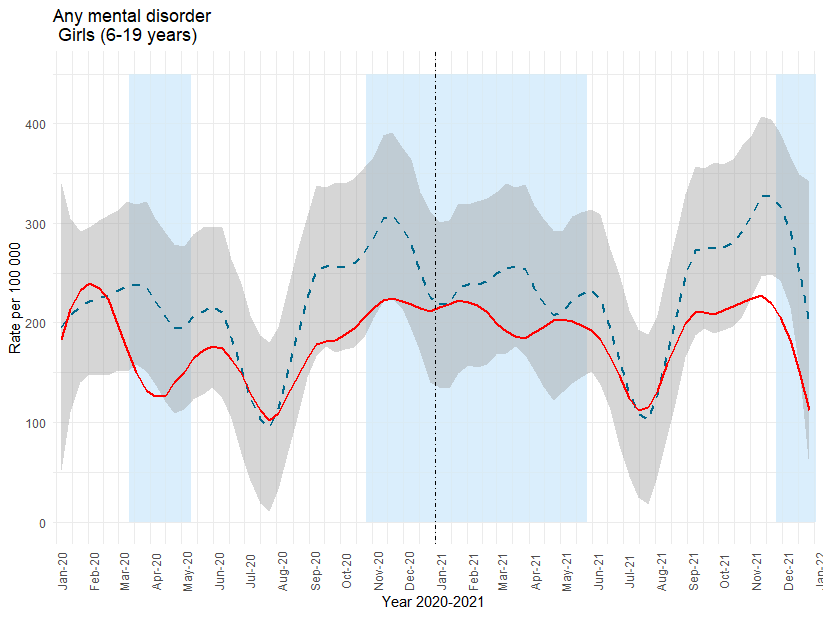

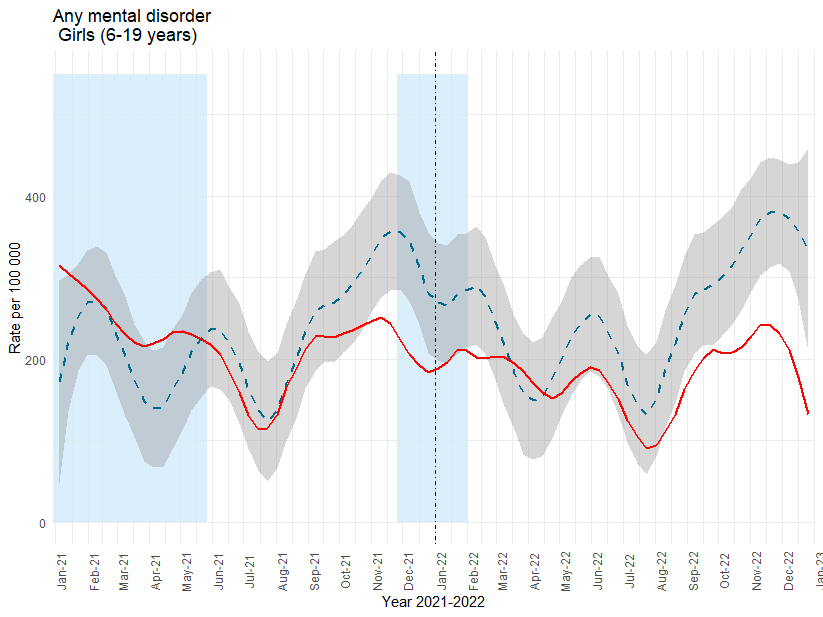


**sFigure 1.** Time series plots for observed weekly consultations per 100.000 (solid red line) for any mental health condition (ICPC-2 codes, chapter P) for boys and girls in primary healthcare. The dashed blue line is the predicted number of consultations, with 99.9% confidence interval in grey. Light blue columns represent periods with strict social distancing measures from the Norwegian government. The left column shows results for inclusion year 2018, with follow-up years 2019-2020. The center column shows results for inclusion year 2019, with follow-up years 2020-2021. The right column shows results for inclusion year 2020, with follow-up years 2021 and 2022. Results for **boys** is shown in the **top row**; **girls** in the **bottom row.**

### **sFigure2**: Anxiety/depression contacts in primary care for boys and girls (ages 6–19) – including 2022


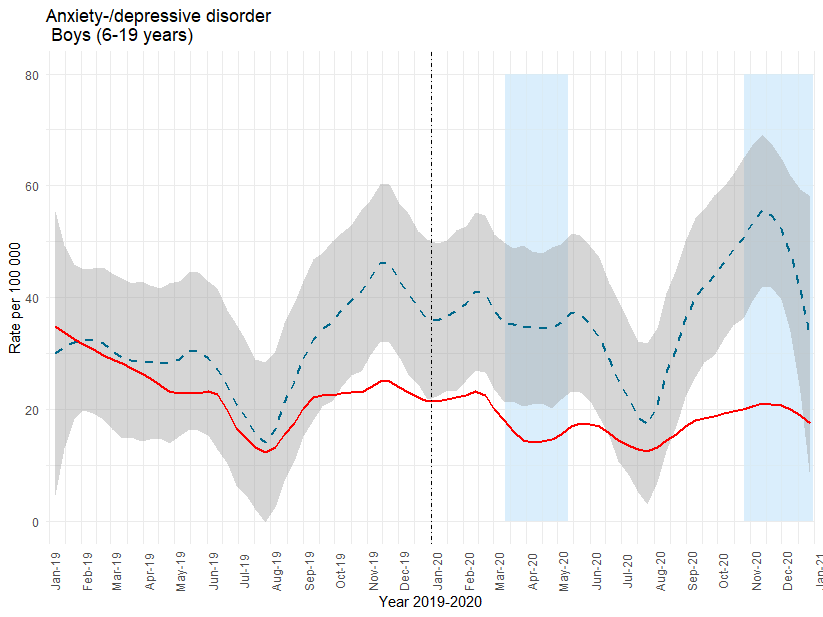

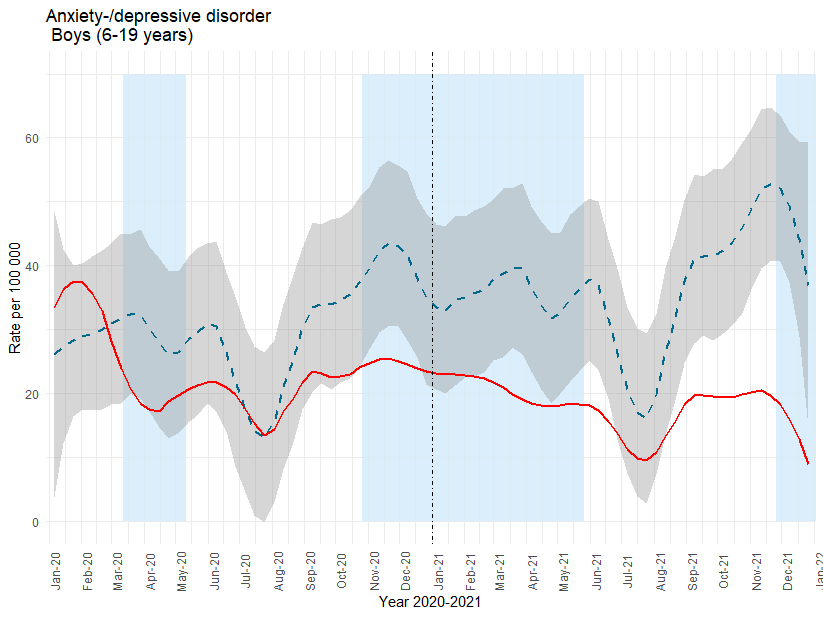

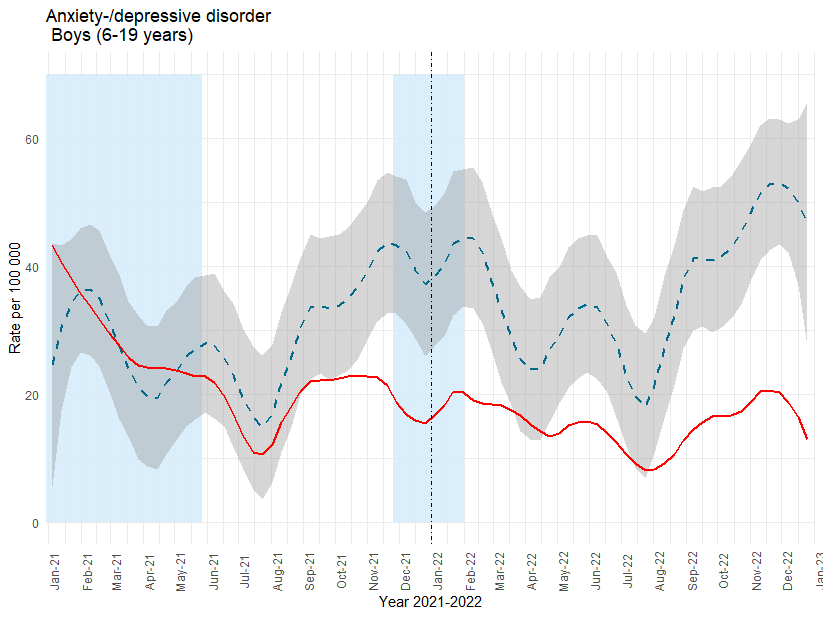


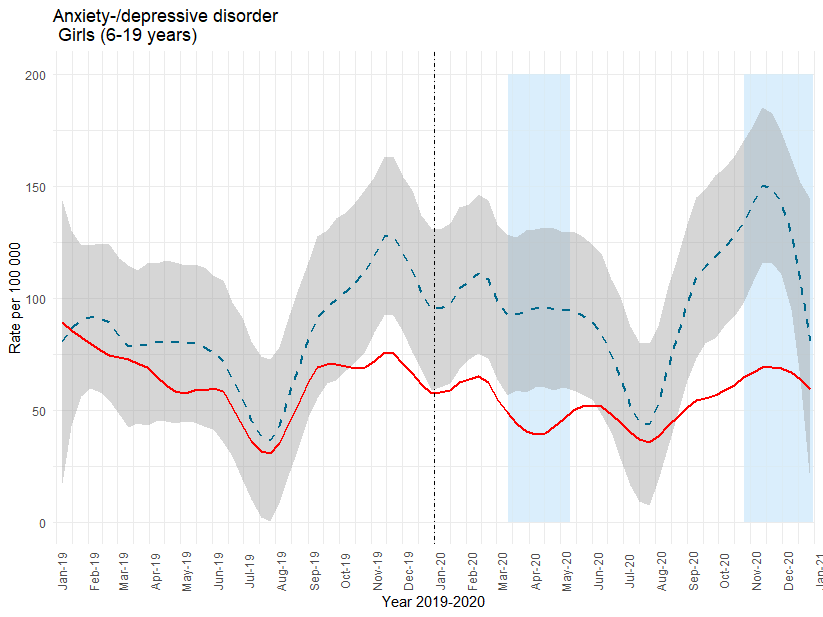

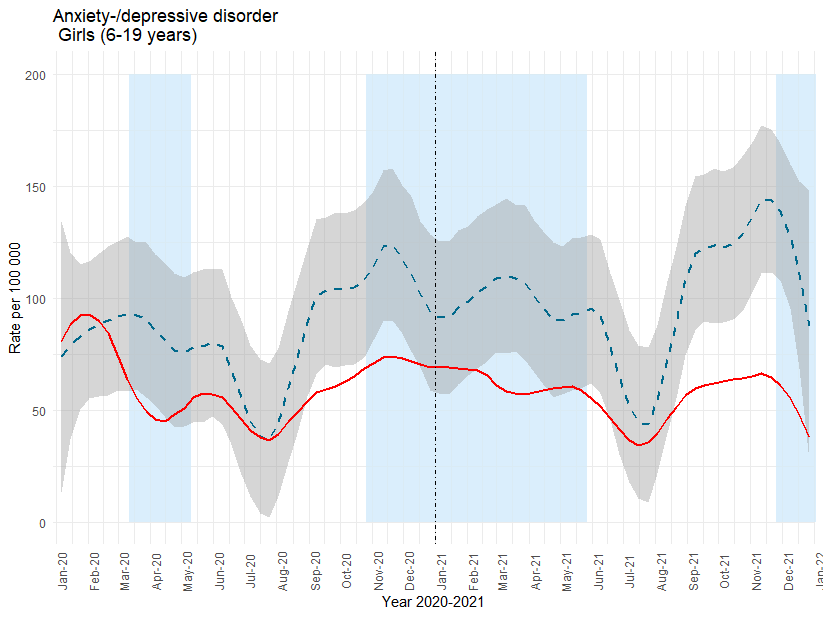

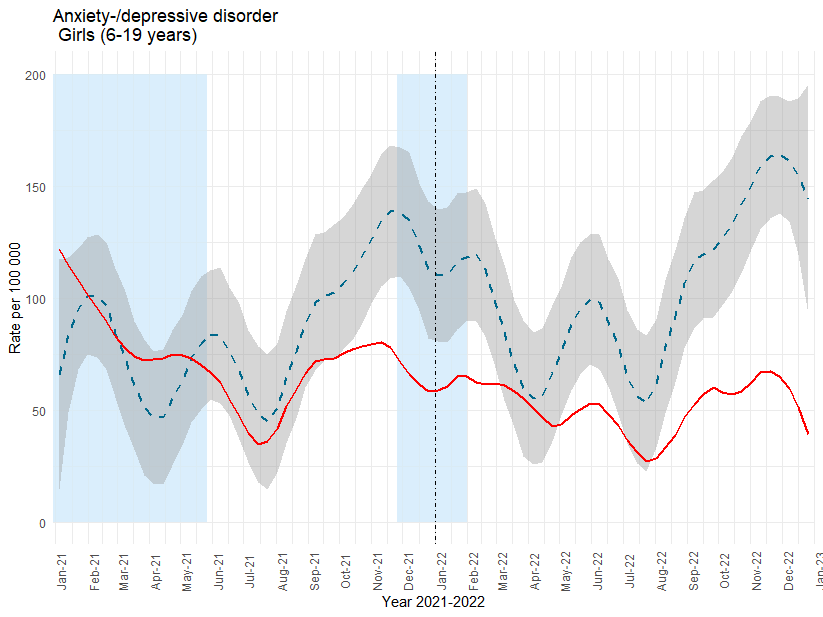


**sFigure 2.** Time series plots for observed weekly consultations per 100.000 (solid red line) for anxiety/depression (ICPC-2 codes P01, P03, P74, P76, P79, P81, P82 for boys and girls in primary healthcare. The dashed blue line is predicted consultations, with 99.9% confidence interval in grey. Light blue columns represent periods with strict social distancing measures from the Norwegian government. The left column shows results for inclusion year 2018, with follow-up years 2019-2020. The center column shows results for inclusion year 2019, with follow-up years 2020-2021. The right column shows results for inclusion year 2020, with follow-up years 2021 and 2022. Results for **boys** is shown in the **top row**; **girls** in the **bottom row.**

### **sFigure3:** ADHD contacts in primary care for boys and girls (ages 6–19) – including 2022

**
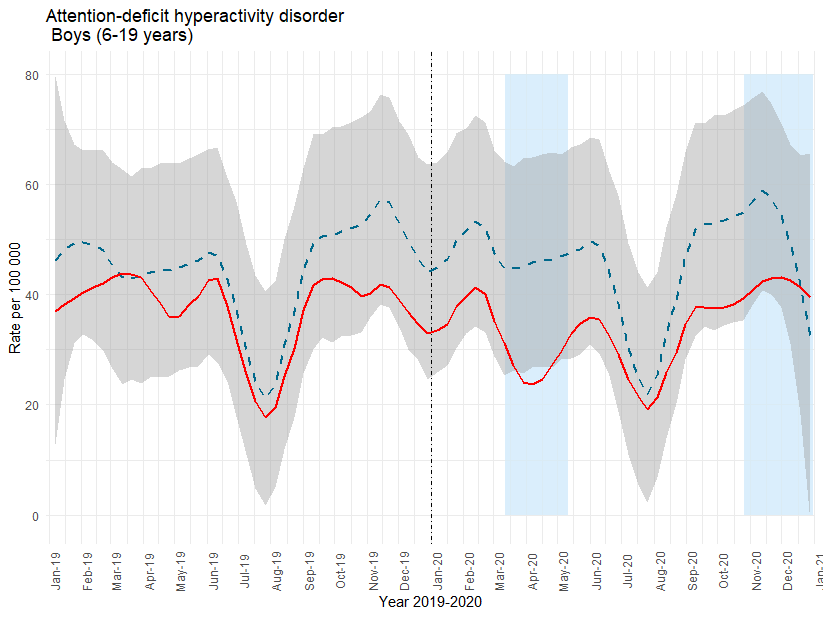

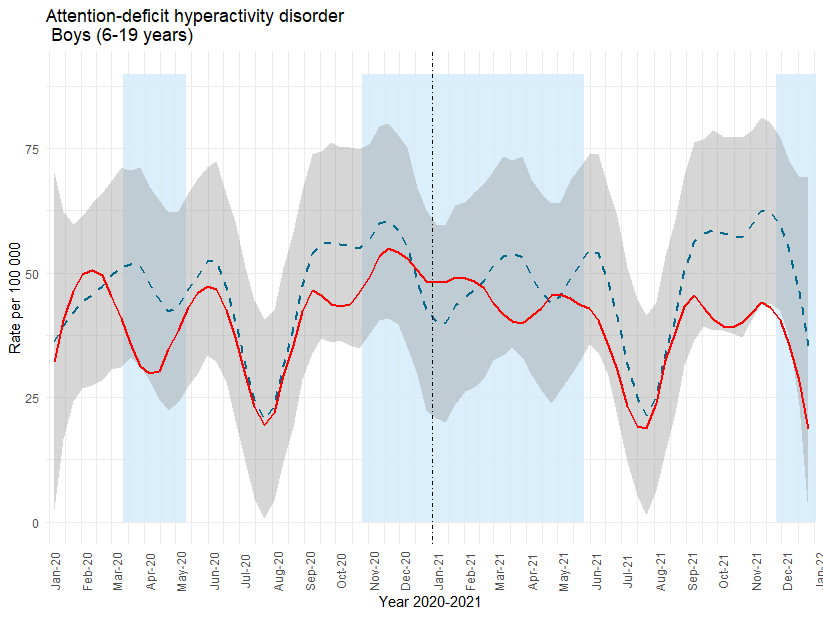

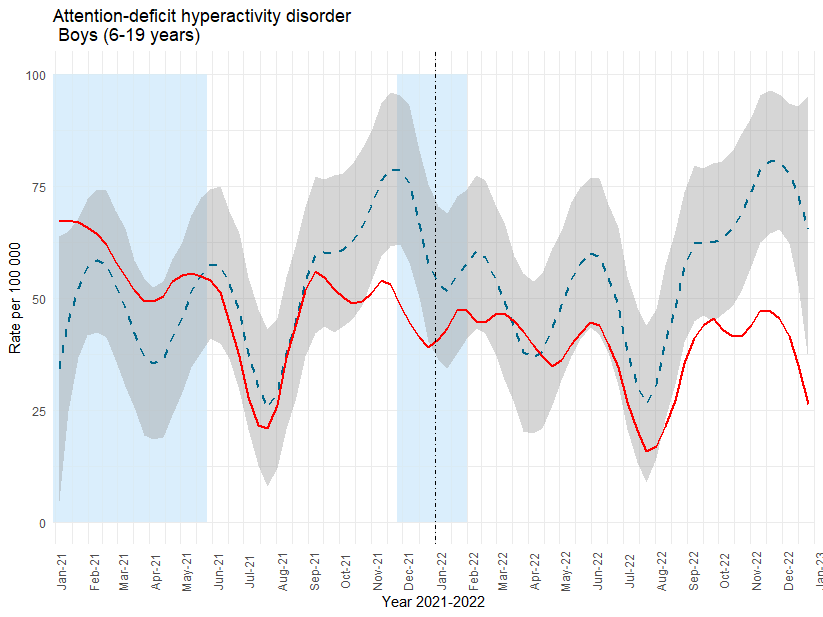
**

**
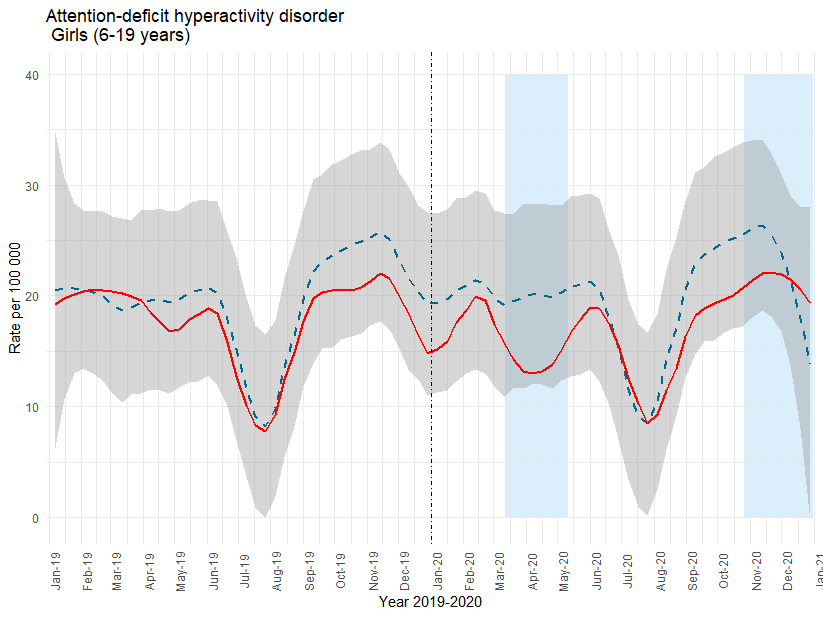

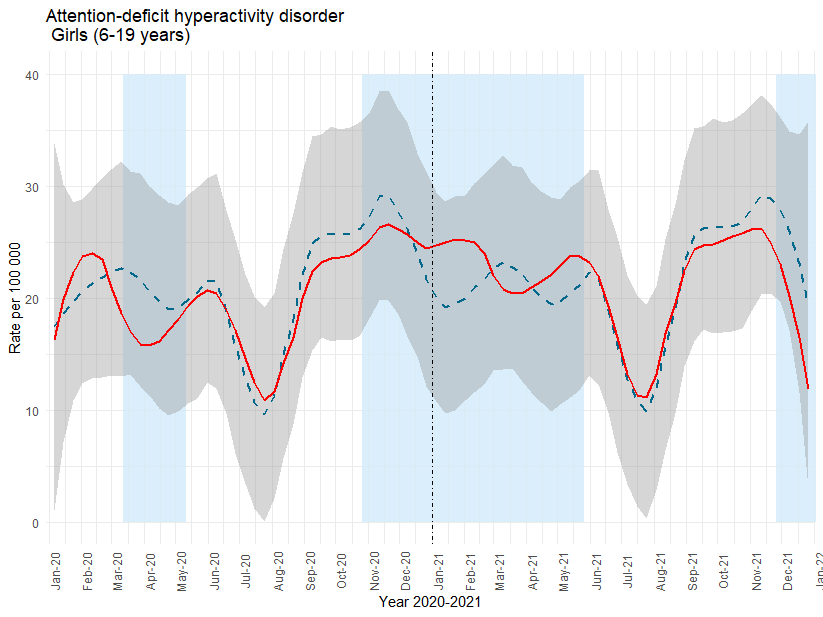

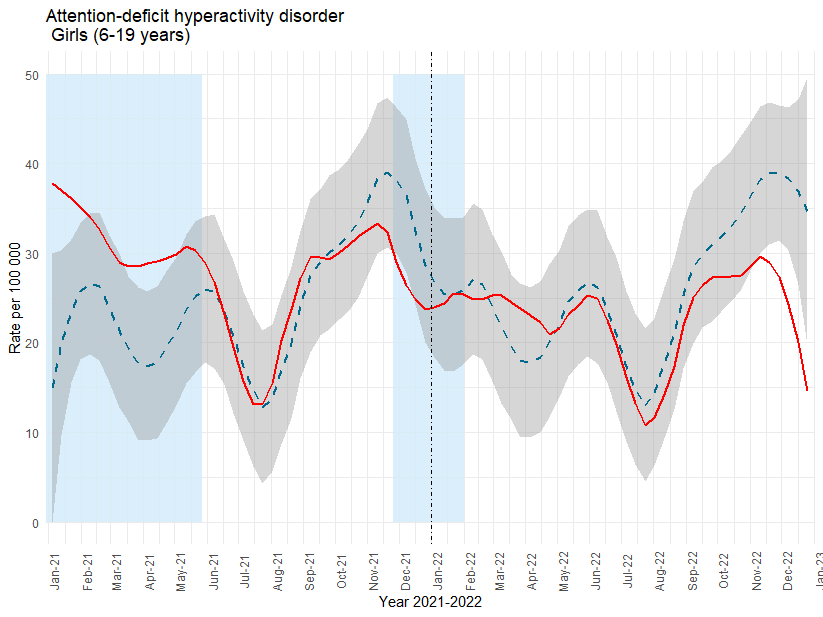
**

**sFigure 3.** Time series plots for observed weekly consultations (solid red line) for ADHD per 100.000 (ICPC-2 code P81) for boys and girls in primary healthcare. The dashed blue line is the predicted consultations, with 99.9% confidence interval in grey. Light blue columns represent periods with strict social distancing measures from the Norwegian government. The left column shows results for inclusion year 2018, with follow-up years 2019-2020. The center column shows results for inclusion year 2019, with follow-up years 2020-2021. The right column shows results for inclusion year 2020, with follow-up years 2021 and 2022. Results for **boys** is shown in the **top row**; **girls** in the **bottom row.**

### **sFigure 4**: Sleep problems in primary care for boys and girls (ages 6–19)

The trend across all three follow-up periods showed that weekly contacts with primary healthcare for sleep problems were well below predicted levels most of the time for both boys (top row) and girls (bottom row), with a similar pattern of lower-than-predicted contacts (sFigure 4).


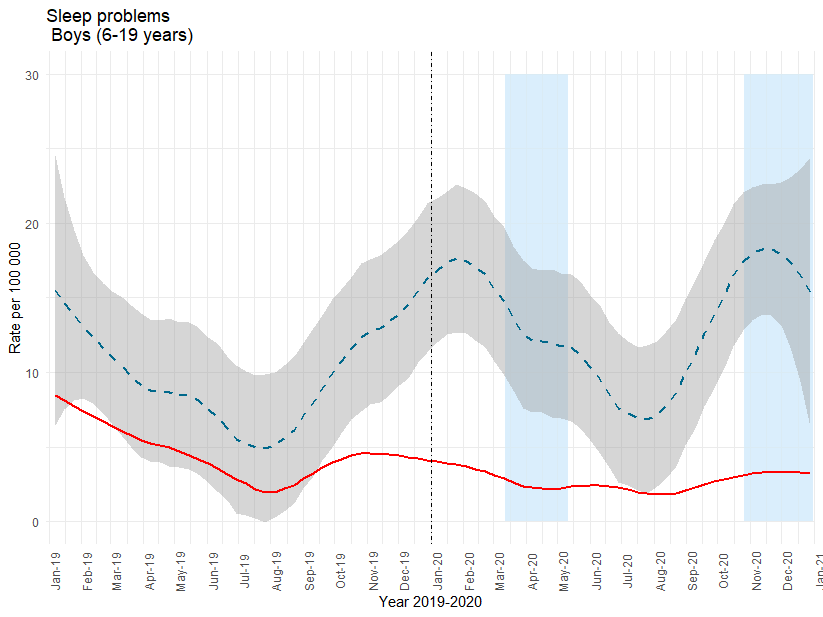

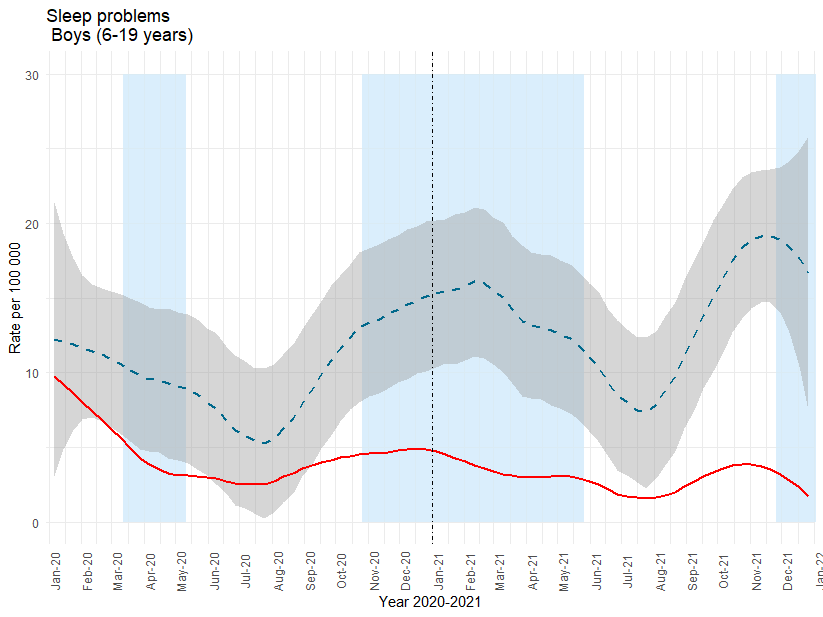

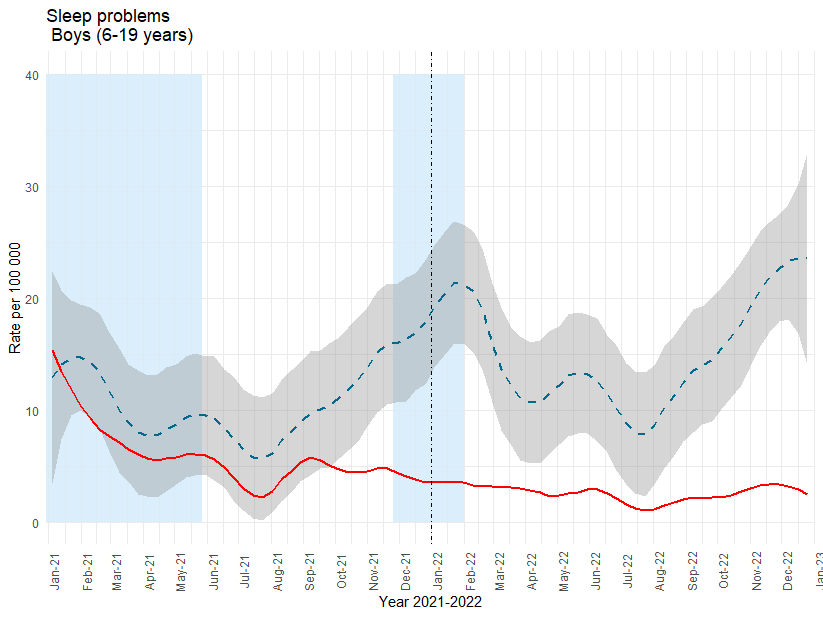


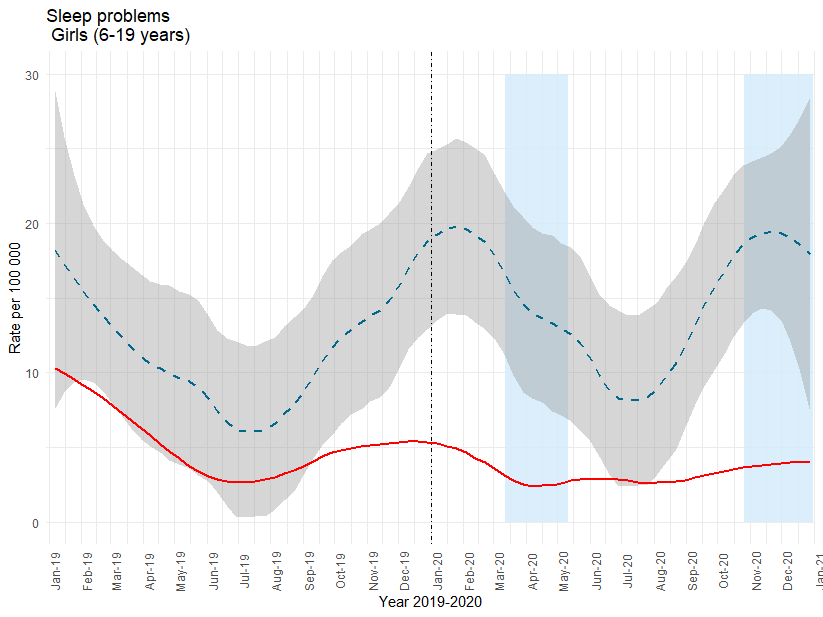

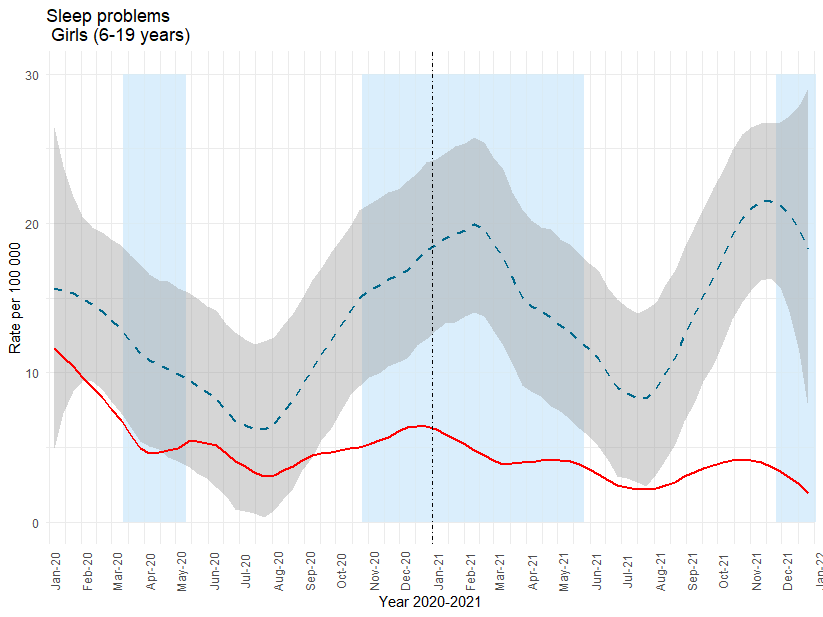

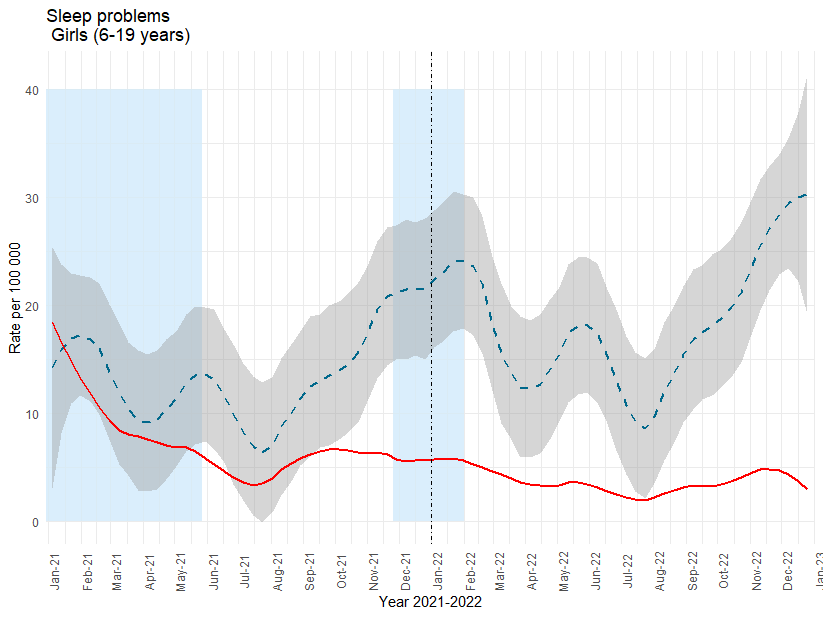


**sFigure 4.** Time series plots for observed weekly consultations per 100.000 (solid red line) for sleep problems in primary care (ICPC-2 code P06) for boys and girls. The dashed blue line is the predicted consultations, with 99.9% confidence interval in grey. Light blue columns represent periods with strict social distancing measures from the Norwegian government. The left column shows results for inclusion year 2018, with follow-up years 2019-2020. The center column shows results for inclusion year 2019, with follow-up years 2020-2021. The right column shows results for inclusion year 2020, with follow-up years 2021 and 2022. Results for **boys** is shown in the **top row**; **girls** in the **bottom row.**

### **sFigure 5**: Autism spectrum diagnoses in specialist care for boys and girls (ages 6–19)

In specialist healthcare for ASD, observed weekly contacts were in line with predictions most of the time in both follow-up periods (sFigure 5). However, there were periods with lower-than-predicted contact levels for both boys (top row) and girls (bottom row), and boys had more prolonged and more pronounced periods with lower-than-predicted contact.


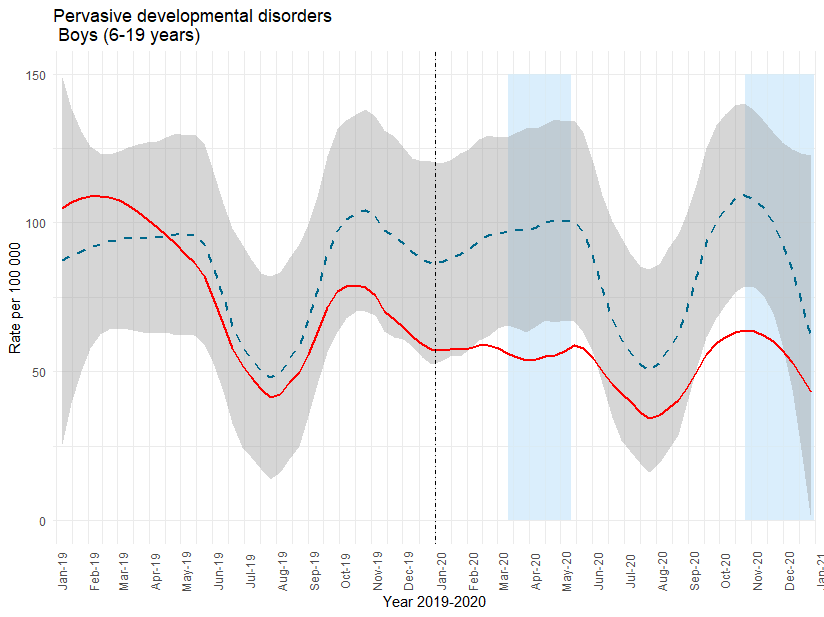

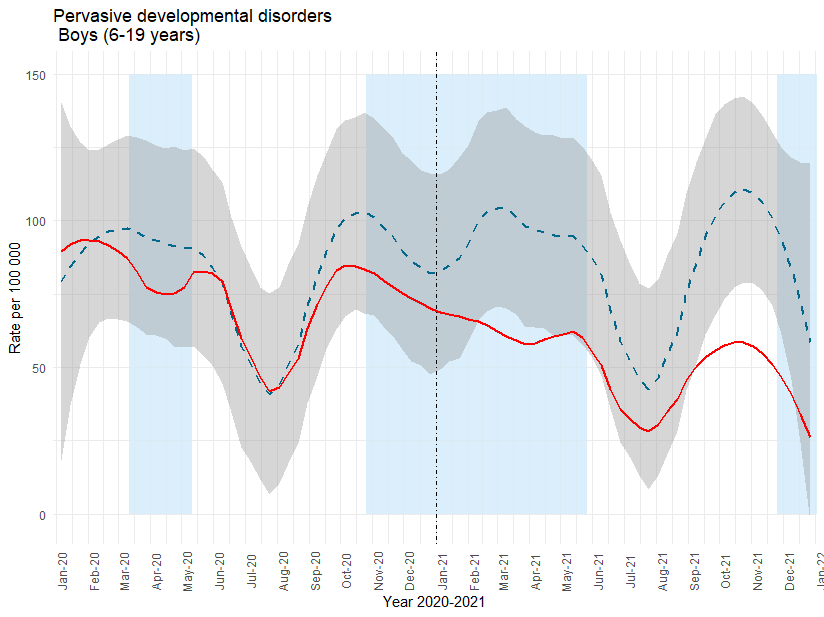


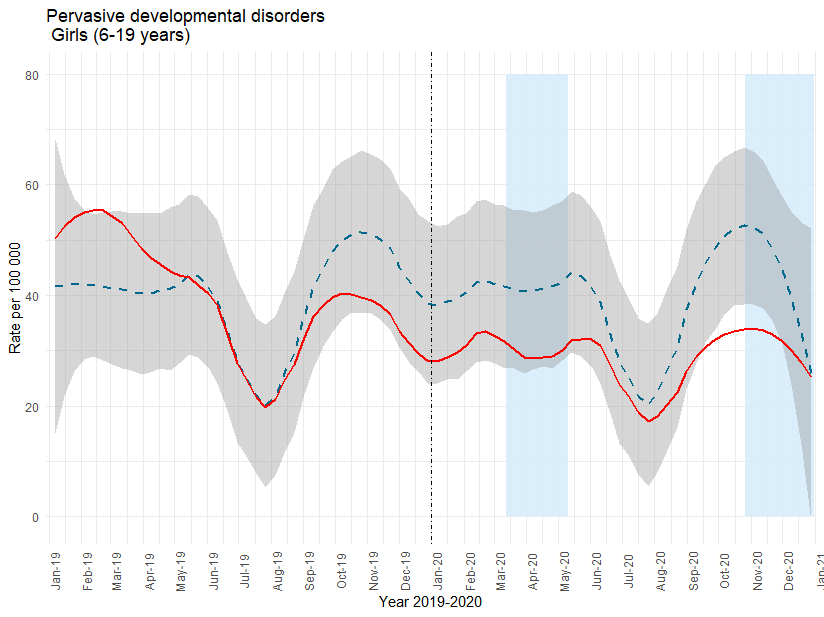

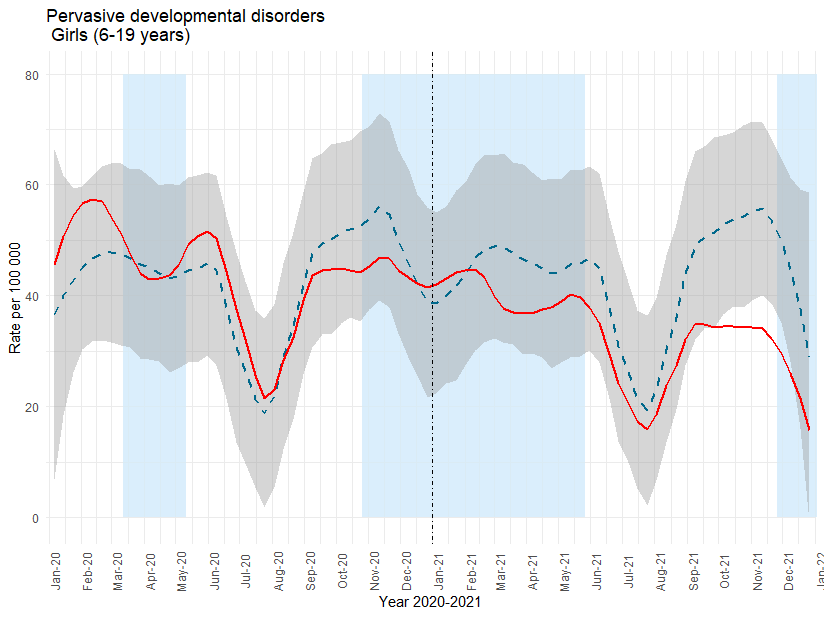


**sFigure 5.** Time series plots for observed weekly consultations per 100.000 (solid red line) for autism spectrum diagnoses in specialist healthcare (ICD-10 code F84) for boys and girls. The dashed blue line is the predicted consultations, with 99.9% confidence interval in grey. Light blue columns represent periods with strict social distancing measures from the Norwegian government. The left column shows results for inclusion year 2018, with follow-up years 2019-2020. The right column shows results for inclusion year 2019, with follow-up years 2020-2021. Results for **boys** is shown in the **top row**; **girls** in the **bottom row**.
